# Pregnanolone Glutamate: A Dual-Fate Delivery System for Neuroactive Steroids in Perinatal Focal Cerebral Ischemia

**DOI:** 10.3390/ijms27052506

**Published:** 2026-03-09

**Authors:** Grygoriy Tsenov, Iqra Bano, Marta Velíková, Viera Kútna, Hana Chodounská, Eva Kudová, Josef Bulant, Martin Hill

**Affiliations:** 1 Preclinical Research Program, Division of Experimental Neurobiology, National Institute of Mental Health, 250 67 Klecany, Czech Republic; grygoriy.tsenov@gmail.com (G.T.); iqra.chandio@nudz.cz (I.B.); viera.kutna@nudz.cz (V.K.); 2 Department of Animal Physiology, Faculty of Science, Charles University, Albertov 6, 128 00 Prague, Czech Republic; 3 Institute of Endocrinology, 110 00 Prague, Czech Republic; mvelikova@endo.cz (M.V.); josef.bulant@vfn.cz (J.B.); 4 Institute of Organic Chemistry and Biochemistry of the Czech Academy of Sciences, 160 00 Prague, Czech Republic; hana.chodounska@uochb.cas.cz (H.C.); eva.kudova@uochb.cas.cz (E.K.); 5 Department of Pediatrics and Inherited Metabolic Disorders, First Faculty of Medicine, Charles University and General University Hospital in Prague, 128 08 Prague, Czech Republic

**Keywords:** pregnanolone glutamate, neuroactive steroids, steroidome, pharmacokinetics, blood-brain barrier, metabolic segregation, neuroprotection, GC-MS/MS

## Abstract

Pregnanolone glutamate (PG) is a synthetic neurosteroid analog showing promise for treating ischemic brain injury, yet its blood–brain barrier (BBB) transport and metabolic fate remain unclear. We investigated the pharmacokinetics of PG in postnatal day 12 rats of both sexes subjected to endothelin-1 (ET-1)-induced focal hippocampal ischemia. Animals received PG (1 mg/kg intraperitoneal (i.p.)) or vehicle; serum and hippocampal steroidomes were profiled 60 min post-administration using gas chromatography-tandem mass spectrometry (GC-MS/MS) (hippocampus: n = 16 PG+, n = 27 PG−; multi-tissue subset: n = 6 PG+, n = 21 PG−). Our data revealed a “dual-fate” mechanism: PG undergoes systemic hydrolysis as a prodrug, as suggested by the tissue distribution pattern at 60 min post-administration, but also crosses the BBB intact, with significant parent conjugate accumulation in the hippocampus (42.3 pmol/g). The brain functioned as a “metabolic sink”, passively accumulating metabolites generated in peripheral organs—such as 17-hydroxypregnanolone—despite local absence of synthesizing enzymes (e.g., CYP17A1). Crucially, PG induced “metabolic segregation” within the central nervous system (CNS): the pharmacological 5β-pathway was saturated (~170-fold pregnanolone increase), while endogenous neuroprotective 5α-pathway (allopregnanolone) homeostasis remained preserved, contrasting with peripheral metabolic saturation. Preferential hippocampal accumulation of 3-oxo and 3β-isomers suggests autonomous regulatory buffering via oxidative 17β-hydroxysteroid dehydrogenase (HSD17B) enzymes, protecting against excessive GABAergic inhibition. This unique pharmacokinetic profile—combining metabolic segregation with active central buffering—defines PG as a dual-mechanism delivery system that generates central neuroactive metabolites—several with previously established GABAergic and neuroprotective activity—without disrupting endogenous neurosteroidogenesis, positioning it as a promising neurotherapeutic candidate minimizing physiological steroid homeostasis disruption. Importantly, the present study characterizes the pharmacokinetic and metabolic fate of PG; the neuroprotective efficacy of PG was demonstrated in our prior functional studies using the same model.

## 1. Introduction

Neurosteroids represent a promising class of neuroprotective agents in ischemic stroke. Estrogen, progesterone, allopregnanolone, and dehydroepiandrosterone (DHEA) exert protective effects through both genomic (intracellular receptor-mediated) and non-genomic (membrane receptor-mediated) pathways [1]. Among these, allopregnanolone—a progesterone metabolite—acts as a potent positive allosteric modulator of GABA_A_R, enhancing chloride channel conductance and neuronal inhibition [1]. Pregnanolone, a structural isomer of allopregnanolone, shares similar GABAergic properties and may exhibit comparable neuroprotective effects in ischemic brain injury.

Perinatal hypoxic-ischemic brain injury represents a critical clinical challenge, often leading to severe long-term neurological sequelae, including cerebral palsy, epilepsy, and cognitive deficits. The pathophysiology of this damage involves a cascade of excitotoxicity, characterized by excessive activation of *N*-methyl-*D*-aspartate receptors (NMDARs), intracellular calcium overload, and subsequent neuroinflammation [2,3,4]. Under physiological conditions, the developing brain possesses endogenous protective mechanisms, primarily mediated by neuroactive steroids (NASs). During late pregnancy, the fetal brain is exposed to high concentrations of progesterone metabolites, such as allopregnanolone (3α,5α-tetrahydroprogesterone) and pregnanolone (3α,5β-tetrahydroprogesterone), which act as potent positive modulators of type A GABA receptor (GABA_A_R) and negative modulators of N-methyl-D-aspartate receptor (NMDAR) [5,6]. However, the precipitous drop in these protective steroid levels immediately after birth leaves the immature brain particularly vulnerable to excitotoxic insults [6,7].

Given the potent neuroprotective properties of these endogenous steroids, their therapeutic application has been extensively explored. Neurosteroids act as positive modulators of GABA_A_R receptors, providing neuroprotection through multiple mechanisms including reduced excitotoxicity and anti-inflammatory effects [8,9,10]. Nevertheless, the clinical use of natural neuroactive steroids is limited by their poor water solubility, rapid metabolic clearance, and low bioavailability when administered systemically [11,12]. Sulfated neurosteroids such as pregnenolone sulfate have been shown to act as positive modulators of NMDA receptors through extracellularly directed sites distinct from other known modulatory sites [13], suggesting that steroid conjugation may represent a general strategy for neuromodulation. However, sulfated steroids exhibit limited blood–brain barrier (BBB) permeability [14,15], prompting the development of alternative conjugates such as glutamate esters. To overcome these pharmacokinetic obstacles, medicinal chemistry has focused on developing conjugated steroid derivatives that improve solubility while retaining neuroactive potency. Among these, pregnanolone glutamate (PG; 20-oxo-5β-pregnan-3α-yl L-glutamyl 1-ester) has emerged as a promising candidate. Previous studies by our group have demonstrated that systemic administration of PG significantly reduces seizure severity and provides neuroprotection in models of focal cerebral ischemia in immature rats [16,17].

**Figure 1:**
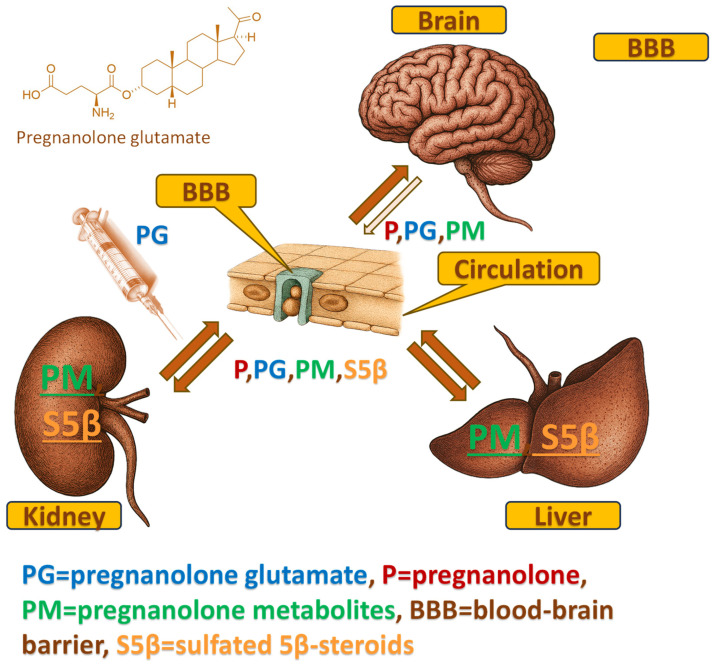
Overview of the systemic disposition, blood–brain barrier (BBB) passage, and organ-specific distribution of pregnanolone glutamate (PG) and its metabolites in the perinatal focal cerebral ischemia (pFCI) model. Following intraperitoneal (i.p.) administration (5 min post-intrahippocampal ET-1 injection), PG enters the circulation and undergoes peripheral biotransformation to pregnanolone (P), downstream pregnanolone metabolites (PM), and sulfated 5β-steroids (S5β), predominantly in the liver and kidney. A fraction of intact PG crosses the BBB, together with P and selected metabolites, resulting in their accumulation within the brain. The schematic illustrates the dual-fate mechanism of PG: (i) peripheral enzymatic hydrolysis generating lipophilic neuroactive derivatives that enter the CNS, and (ii) direct BBB transport of intact PG. Abbreviations: PG, pregnanolone glutamate; P, pregnanolone; PM, pregnanolone metabolites; BBB, blood–brain barrier; S5β, sulfated 5β-reduced steroids.

Despite these promising functional outcomes, the precise pharmacokinetic mechanism underlying the action of PG remains a "black box". As a polar conjugate, PG was originally designed to improve solubility, yet its polarity theoretically hinders passive diffusion across the blood–brain barrier (BBB) (Table 1). This raises a fundamental question: Does PG utilize a specific transport mechanism to cross the BBB intact, or does it serve primarily as a peripheral prodrug requiring biotransformation to exert its central effects? Furthermore, the potential interaction between this exogenous 5β-steroid and the endogenous 5β-steroidogenic pathway—crucial for maintaining natural neuroprotection—has not been fully characterized. To illustrate the working hypothesis of PG as a peripheral shuttle—undergoing rapid systemic hydrolysis, entering metabolic organs, and partially crossing the BBB intact—we provide a schematic overview of its proposed distribution pathways (Figure 1).

**Figure 2:**
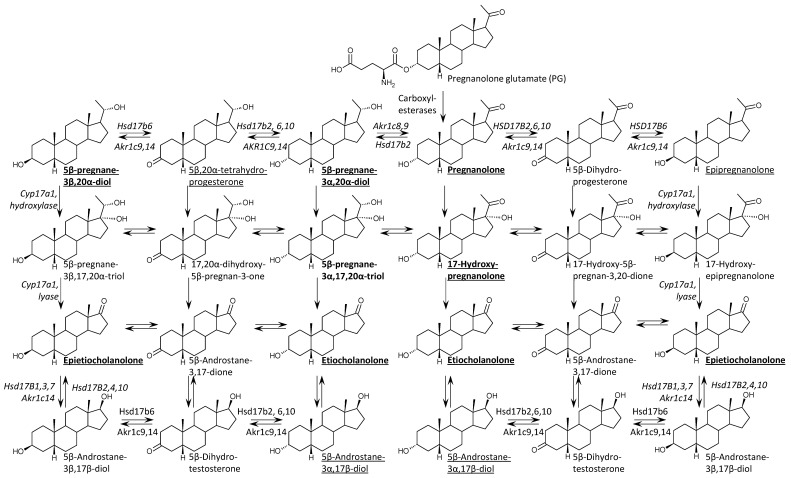
Metabolic scheme illustrating enzymatic conversions of pregnanolone glutamate (PG) to pregnanolone and further 5β-reduced metabolites. Bold labels denote quantified free steroids, while underlined labels indicate quantified conjugated steroids. Enzymes involved in individual steps (e.g., carboxylesterases, aldo-keto reductase family 1 member C (AKR1C) isoforms, 17β-hydroxysteroid dehydrogenase (HSD17B) isoforms, CYP17A1 hydroxylase/lyase) are shown next to corresponding reactions.

Using advanced gas chromatography-tandem mass spectrometry (GC-MS/MS), we performed comprehensive steroidome profiling in serum and hippocampal tissue, as well as in the liver and kidneys as key metabolic organs, following intraperitoneal PG administration. By analyzing the distribution of specific metabolites and leveraging the known tissue-specific expression of steroidogenic enzymes (such as the negligible CYP17A1 and SULT2A1 activity in the brain), we investigated whether the neuroactive pool in the CNS originated from local metabolism or peripheral transport. We hypothesize that PG functions as a “dual-fate” delivery system, utilizing systemic metabolism to generate a spectrum of lipophilic metabolites while simultaneously crossing the BBB via carrier-mediated transport, thereby selectively enhancing the 5β-signaling pathway without disrupting endogenous neurosteroidogenesis (Figure 2). To test this hypothesis, we compared steroidome profiles between the PG−treated animals (PG+, n = 16) and vehicle-treated controls (PG−, n = 27) at 60 min post-administration.

## 2. Results

### 2.1. Comparison of Metabolic Profiles of 5β and 5α Steroids in Serum, Right Hippocampus, Liver and Kidney Between PG+ and PG− Rats

#### 2.1.1. Concentrations of 5β-Reduced Steroids in the Right Hippocampus (Table 2)

Table 2 presents the concentrations of 5β-reduced steroids in the ischemic right hippocampus (RH, see Section 6.3) of postnatal day 12 (P12) rats following the intraperitoneal administration of pregnanolone glutamate (PG). The enzymatic pathways responsible for the generation of these metabolites are illustrated in Figure 2. The administration of PG resulted in a statistically significant increase in the levels of the parent compound, pregnanolone, which rose from a basal mean of 0.41 pmol/g in PG− rats to 69.3 pmol/g in the PG+ group (*p* < 0.001). A significant elevation was also observed for the conjugated form of pregnanolone, reaching 42.3 pmol/g compared to 0.085 pmol/g in PG− animals (*p* < 0.001). Regarding downstream metabolites, the 17-hydroxypregnanolone levels increased to 11.4 pmol/g, and the 5β-pregnane-3α,20α-diol concentrations rose to 12.8 pmol/g compared to 1.71 pmol/g in the PG− rats (*p* < 0.001). Etiocholanolone and its conjugate also exhibited statistically significant increases (*p* < 0.01). In contrast, the levels of epipregnanolone conjugates did not show a statistically significant difference compared to the PG− rats (*p* = 0.087). Free epipregnanolone, measured in the tissue distribution subset (Table 3), exhibited high inter-individual variability (coefficient of variation (CV) ≈82%) and no significant difference between groups (214 vs. 132 fmol/g, *p* > 0.05), and was therefore excluded from the main hippocampal analysis (Table 2). Two-way ANOVA analysis confirmed a significant main effect of PG treatment, whereas no significant main effect of sex was observed for the majority of the analyzed 5β-steroids (Table 2).

#### 2.1.2. Relative Abundance of 5β-Reduced Steroids in the Right Hippocampus (Table 4)

Beyond the absolute concentrations, the analysis of the relative abundance of individual 5β-steroids revealed a fundamental shift in the metabolic profile (Table 4). In PG− animals, the steroidome was dominated by downstream metabolites, such as 5β-pregnane-3β,20α-diol (~35%) and 5β-pregnane-3α,20α-diol (~23%). However, the administration of PG resulted in a redistribution of the pool towards the administered precursors. In the PG+ group, pregnanolone and its conjugate collectively accounted for approximately 71% of the total 5β-steroid content, confirming the robust saturation of the initial steps of the metabolic pathway.

#### 2.1.3. Concentrations of Endogenous 5α-Steroids in the Right Hippocampus (Table 5)

Table 5 summarizes the concentrations of endogenous 5α-reduced steroids in the right hippocampus. No statistically significant differences were observed in the levels of free allopregnanolone between PG+ rats (2.96 pmol/g) and PG− rats (3.69 pmol/g; *p* = 0.411). Similarly, the levels of the precursor 5α-dihydroprogesterone did not differ significantly between the groups (*p* = 0.253). In contrast, the concentrations of conjugated metabolites exhibited significant increases following PG administration. Conjugated allopregnanolone levels rose from 0.739 pmol/g in PG− rats to 1.35 pmol/g in the PG+ group (*p* < 0.01). Significant elevations were also observed for conjugated 5α-pregnane-3α,20α-diol (*p* < 0.05) and conjugated androsterone (*p* < 0.001). Two-way ANOVA revealed a significant main effect of sex for 5α-dihydroprogesterone (*p* = 0.026), allopregnanolone (*p* = 0.033), and 17-hydroxyallopregnanolone (*p* = 0.008).

#### 2.1.4. Tissue-Specific Distribution of 5β-Steroids (Figure 3)

**Figure 3:**
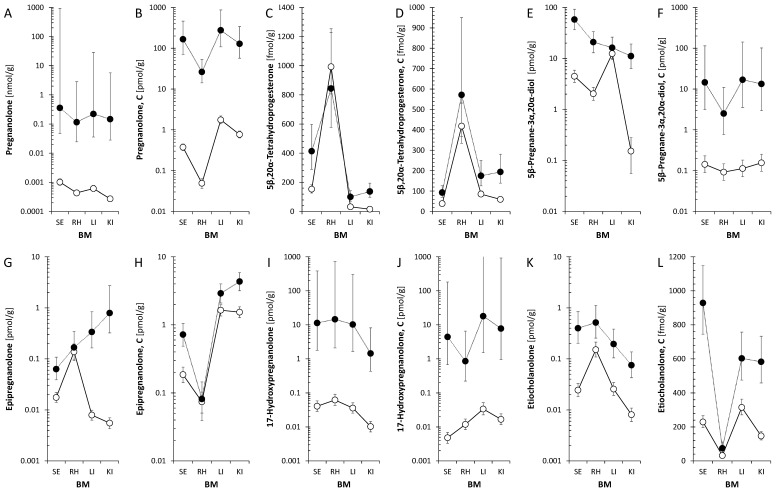
Distribution of pregnanolone, 5β-pregnane-3α,20α-diol, and downstream metabolites (5β,20α-tetrahydroprogesterone, epipregnanolone) across serum, hippocampus, liver, and kidney following PG administration. Multi-tissue analysis performed on a representative subset (n = 6 PG+, n = 21 PG−) optimized for peripheral metabolism profiling. Conjugated forms were detected in all tissues, with the highest levels in the liver and kidney. Epipregnanolone conjugates were enriched in the kidney (median > 4.9 pmol/g) compared to the hippocampus (~58 fmol/g). Two-way ANOVA confirmed significant PG × BM interactions for most metabolites (*p* < 0.001). Panel (**A**). PG: F = 290.5, *p* < 0.001, ηp^2^ = 0.888; BM: F = 6.2, *p* < 0.001, ηp^2^ = 0.202; PG × BM: F = 3.6, *p* = 0.017, ηp^2^ = 0.129; Subj(PG): F = 2, *p* = 0.013, ηp^2^ = 0.404; Panel (**B**). PG: F = 703.2, *p* < 0.001, ηp^2^ = 0.952; BM: F = 80.9, *p* < 0.001, ηp^2^ = 0.771; PG × BM: F = 24.8, *p* < 0.001, ηp^2^ = 0.508; Subj(PG): F = 2, *p* = 0.011, ηp^2^ = 0.411; Panel (**C**). PG: F = 63.8, *p* < 0.001, ηp^2^ = 0.56; BM: F = 169.6, *p* < 0.001, ηp^2^ = 0.874; PG × BM: F = 18.7, *p* < 0.001, ηp^2^ = 0.435; Subj(PG): F = 1.5, *p* = 0.111, ηp^2^ = 0.332; Panel (**D**). PG: F = 32.7, *p* < 0.001, ηp^2^ = 0.497; BM: F = 76.6, *p* < 0.001, ηp^2^ = 0.761; PG × BM: F = 5.4, *p* = 0.002, ηp^2^ = 0.183; Subj(PG): F = 2.2, *p* = 0.006, ηp^2^ = 0.428; Panel (**E**). PG: F = 107.1, *p* < 0.001, ηp^2^ = 0.736; BM: F = 37.3, *p* < 0.001, ηp^2^ = 0.609; PG × BM: F = 18.9, *p* < 0.001, ηp^2^ = 0.44; Subj(PG): F = 1.9, *p* = 0.019, ηp^2^ = 0.397; Panel (**F**). PG: F = 206.2, *p* < 0.001, ηp^2^ = 0.686; BM: F = 1.9, *p* = 0.137, ηp^2^ = 0.0706; PG × BM: F = 0.5, *p* = 0.674, ηp^2^ = 0.0201; Subj(PG): F = 0.8, *p* = 0.738, ηp^2^ = 0.209; Panel (**G**). PG: F = 98.8, *p* < 0.001, ηp^2^ = 0.795; BM: F = 21.1, *p* < 0.001, ηp^2^ = 0.468; PG × BM: F = 47.7, *p* < 0.001, ηp^2^ = 0.665; Subj(PG): F = 2.8, *p* < 0.001, ηp^2^ = 0.492; Panel (**H**). PG: F = 36.3, *p* < 0.001, ηp^2^ = 0.052; BM: F = 183.4, *p* < 0.001, ηp^2^ = 0.385; PG × BM: F = 6.0, *p* = 0.001, ηp^2^ = 0.0199; Subj(PG): F = 1.3, *p* = 0.176, ηp^2^ = 0.0363; Panel (**I**). PG: F = 155.9, *p* < 0.001, ηp^2^ = 0.806; BM: F = 9.1, *p* < 0.001, ηp^2^ = 0.266; PG × BM: F = 1.2, *p* = 0.336, ηp^2^ = 0.0438; Subj(PG): F = 2, *p* = 0.011, ηp^2^ = 0.4; Panel (**J**). PG: F = 203.8, *p* < 0.001, ηp^2^ = 0.803; BM: F = 6.6, *p* < 0.001, ηp^2^ = 0.208; PG × BM: F = 3.3, *p* = 0.025, ηp^2^ = 0.116; Subj(PG): F = 1.5, *p* = 0.091, ηp^2^ = 0.334; Panel (**K**). PG: F = 88.4, *p* < 0.001, ηp^2^ = 0.653; BM: F = 32.3, *p* < 0.001, ηp^2^ = 0.567; PG × BM: F = 4.2, *p* = 0.008, ηp^2^ = 0.146; Subj(PG): F = 1.6, *p* = 0.07, ηp^2^ = 0.347; Panel (**L**). PG: F = 275, *p* < 0.001, ηp^2^ = 0.689; BM: F = 112.1, *p* < 0.001, ηp^2^ = 0.824; PG × BM: F = 9.2, *p* < 0.001, ηp^2^ = 0.277; Subj(PG): F = 0.6, *p* = 0.942, ηp^2^ = 0.165. Abbreviations: SER, serum; H, hippocampus; LI, liver; KI, kidney; BM, biological matrix (tissue type); PG, pregnanolone treatment factor; Subj(PG), subjects nested within PG group; C denotes the conjugated form of the respective analyte; F, Fisher’s F-statistic; *p*, *p*-value; ηp^2^, partial eta-squared (effect size). Black and white dots symbolize PG+ and PG− rats.

Distribution profiles of 5β-steroids in serum, hippocampus, liver, and kidney are shown in Figure 3. This multi-tissue analysis was performed on a representative subset (n = 6 PG+, n = 21 PG−) with complete collection of all biological materials, where large effect sizes for peripheral metabolism allowed for adequate statistical power for detecting tissue-specific differences. Free pregnanolone followed the gradient Serum > Liver > Kidney > Hippocampus, with median hippocampal levels ~107 pmol/g. Conjugated pregnanolone was present in all tissues, with the highest levels in the liver and kidney. 5β-Pregnane-3α,20α-diol, 5β,20α-tetrahydroprogesterone, and epipregnanolone were also detected. Epipregnanolone conjugates were enriched in the kidney (median > 4.9 pmol/g) versus the hippocampus (~58 fmol/g). Two-way ANOVA revealed significant PG × BM interactions for most metabolites (*p* < 0.001).

#### 2.1.5. Quantitative Systemic Distribution of 5β and 5α Steroids (Table 5 and Table 6)

Table 5 and Table 6 detail the quantitative comparison of 5β and 5α steroid levels across serum, hippocampus, liver, and kidney in a representative subset with complete multi-tissue collection (n = 6 PG+, n = 21 PG−), where large effect sizes for peripheral metabolism were expected based on preliminary data, allowing for adequate statistical power without multi-tissue collection from the full cohort. Two-way ANOVA revealed a significant main effect of PG treatment (*p* < 0.001) and Biological Material (*p* < 0.001) for the majority of the analyzed analytes. For the parent compound, pregnanolone, the highest median concentration in the PG+ group was observed in serum (404 pmol/g), followed by the liver (210 pmol/g) and kidney (159 pmol/g). The median concentration in the hippocampus was 107 pmol/g, compared to 0.422 pmol/g in the PG− group. Bonferroni multiple comparisons indicated that the hippocampal levels were significantly lower than the serum levels. Regarding conjugated metabolites, epipregnanolone conjugate levels were 4990 fmol/g in the kidney and 2800 fmol/g in the liver compared to 764 fmol/g in serum and 58.6 fmol/g in the hippocampus. Post hoc analysis showed that concentrations in the kidney and liver were significantly higher than in serum. In the case of 17-hydroxypregnanolone, the concentration in the hippocampus (16 pmol/g) was not statistically different from the serum levels (11.7 pmol/g; *p* > 0.05) but was significantly higher than the concentration in the kidney (2.35 pmol/g).

Readers may note that hippocampal pregnanolone concentrations differ between Table 2 (69.3 pmol/g, geometric mean, n = 16) and Table 3 (107 pmol/g, median, n = 6). This discrepancy arises from three methodological factors: (1) different sample sizes—Table 2 analyzed the full cohort optimized for hippocampal profiling, while Table 3 analyzed a subset with complete multi-tissue collection; (2) different statistical measures—retransformed means (Table 2) versus medians (Table 3); and (3) biological variability in peripheral metabolism. Steroidomic data typically exhibit right-skewed distributions, where median values may exceed geometric means when a subset of high-responder animals is analyzed. Importantly, both measures demonstrate the same fundamental finding: PG administration produced a ~170-fold increase in hippocampal pregnanolone compared to the vehicle controls (0.41 pmol/g in PG− rats, Table 2). The choice of subset analysis for Table 3 was justified by the expectation of large effect sizes in peripheral organs (liver, kidney), allowing for adequate statistical power without requiring multi-tissue collection from all animals. The observed concentration range (69–107 pmol/g) fell well within the expected biological variability for this age group and route of administration, and did not affect the interpretation of the dual-fate delivery mechanism.

**Table 2:** Concentrations of 5β-reduced steroids in the right hippocampus (RH) of PG+ rats compared to PG− rats.

Steroid	Unit	Pregnanolone Glutamate (PG)		Sex		ANOVA (*p*-Value)		
						PG		Sex
		PG+	PG−	Female	Male	*p*	q (FDR)	
Pregnanolone	pmol/g	69.3 (57.4, 83.4) ***	0.41 (0.33, 0.509)	7.05 (5.79, 8.56)	6.79 (5.55, 8.27)	<0.001	<0.001	0.842
Pregnanolone, C	pmol/g	42.3 (35.7, 49.8) ***	0.0845 (0.0577, 0.121)	4.07 (3.27, 5.04)	4.19 (3.35, 5.19)	<0.001	<0.001	0.897
Epipregnanolone, C	fmol/g	128 (84.8, 193)	67.4 (48.9, 92.8)	82.8 (57.7, 119)	104 (72.4, 150)	0.087	0.109	0.515
5β,20α-Tetrahydroprogesterone, C	fmol/g	307 (248, 382)	380 (321, 451)	373 (309, 453)	312 (258, 379)	0.279	0.299	0.342
5β-Pregnane-3α,20α-diol	pmol/g	12.8 (10.4, 15.6) ***	1.71 (1.36, 2.13)	5.51 (4.47, 6.74)	4.72 (3.79, 5.84)	<0.001	<0.001	0.457
5β-Pregnane-3α,20α-diol, C	pmol/g	6.58 (4.98, 8.61) ***	0.135 (0.0922, 0.195)	1.16 (0.847, 1.57)	1.33 (0.973, 1.8)	<0.001	<0.001	0.647
5β-Pregnane-3β,20α-diol	pmol/g	2.66 (2.18, 3.2)	2.8 (2.4, 3.22)	2.71 (2.27, 3.18)	2.75 (2.31, 3.23)	0.775	0.775	0.917
5β-Pregnane-3β,20α-diol, C	pmol/g	1.18 (0.911, 1.51)	1.01 (0.816, 1.23)	0.988 (0.778, 1.24)	1.2 (0.957, 1.49)	0.481	0.481	0.375
17-Hydroxypregnanolone	fmol/g	11,400 (9750, 13,400) ***	38.4 (29.3, 49.9)	1080 (888, 1300)	1150 (947, 1390)	<0.001	<0.001	0.721
17-Hydroxypregnanolone, C	fmol/g	3840 (2510, 5920) ***	13 (10.1, 16.8)	163 (119, 226)	209 (150, 292)	<0.001	<0.001	0.440
5β-Pregnane-3α,17,20α-triol	fmol/g	470 (371, 589) ***	43.6 (32.8, 57.2)	175 (137, 222)	150 (116, 193)	<0.001	<0.001	0.527
Etiocholanolone	fmol/g	500 (344, 715) **	192 (140, 262)	357 (255, 494)	275 (194, 387)	0.008	0.013	0.433
Etiocholanolone, C	fmol/g	339 (203, 576) ***	57.1 (40, 82.1)	118 (77.4, 181)	157 (102, 245)	<0.001	<0.001	0.495
Epietiocholanolone	fmol/g	120 (75.5, 185)	193 (140, 261)	171 (118, 243)	137 (91.5, 198)	0.218	0.252	0.536
Epietiocholanolone, C	fmol/g	177 (124, 255) *	89.6 (68.9, 117)	112 (82.9, 152)	140 (103, 193)	0.035	0.049	0.463
5β-Androstane-3α,17β-diol, C	fmol/g	279 (201, 374) *	149 (110, 196)	194 (142, 258)	221 (161, 293)	0.044	0.056 †	0.659

Note: Data are presented as retransformed means with 95% confidence intervals; median values in multi-tissue subset (Table 5) may differ due to sample size and right-skewed distribution. Relationships were evaluated by two-way ANOVA (factors: PG status and Sex). PG (1 mg/kg) was administered i.p. 5 min after intrahippocampal ET-1 injection; samples were collected 60 min post-injection. n = 16 (PG+), n = 27 (PG− rats). C = conjugated steroid. * *p* < 0.05, ** *p* < 0.01, *** *p* < 0.001 (nominal). Benjamini–Hochberg False Discovery Rate (FDR)-adjusted q-values are reported in the “q (FDR)” column. † q ≥ 0.05: exploratory finding requiring independent confirmation.

**Table 3:** Quantitative distribution of 5β-steroids across serum, hippocampus, liver, and kidney in PG+ rats versus PG− rats.

Steroid	PG	Biological Material (BM)					ANOVA (*p*-Value)			
		Serum (S)	Right Hippocampus (H)	Liver (L)	Kidney (K)	Bonferroni Multiple Comparisons (*p* < 0.05)	PG	BM	PG × BM	Subject
Pregnanolone	−	0.892 (0.816, 1.4)	0.422 (0.313, 0.68)	0.539 (0.364, 1.24)	0.286 (0.155, 0.383)	H < S, L < S, K < S, K < H, K < L	<0.001	<0.001	0.017	0.013
[pmol/g]	+	404 (309, 451) *	107 (98.8, 124) *	210 (135, 513) *	159 (98.4, 213) *	n.s.				
Pregnanolone, C	−	0.39 (0.282, 0.504)	0.0452 (0.0225, 0.0802)	1.43 (1.2, 1.54)	0.832 (0.59, 1.06)	H < S, L > S, K > S, L > H, K > H, K < L	<0.001	<0.001	<0.001	0.011
[pmol/g]	+	184 (140, 200) *	27.6 (22.1, 32.9) *	316 (209, 337) *	150 (134, 179) *	H < S, L > H, K > H				
Epipregnanolone	−	14.5 (9.7, 31.3)	132 (74.5, 368)	6.41 (4.31, 14.5)	6.14 (5.04, 7.06)	H > S, L < S, K < S, L < H, K < H	<0.001	<0.001	<0.001	<0.001
[fmol/g]	+	75.7 (36.9, 124) *	214 (111, 347)	324 (226, 787) *	694 (510, 1960) *	L > S, K > S				
Epipregnanolone, C	−	170 (137, 220)	52.6 (31.8, 121)	1590 (1320, 2050)	1690 (1460, 2100)	H < S, L > S, K > S, L > H, K > H	<0.001	<0.001	0.001	0.176
[fmol/g]	+	764 (654, 897) *	58.6 (18.6, 208)	2800 (2540, 3600) *	4990 (3500, 6370) *	H < S, L > S, K > S, L > H, K > H				
17-Hydroxypregnanolone [fmol/g]	−	44.9 (20.8, 67.3)	62.7 (33.5, 91.1)	68.3 (7.59, 102)	8.75 (7.02, 16.9)	K < S, K < H, K < L	<0.001	<0.001	0.336	0.011
	+	11,700 (10,500, 13,700) *	16,000 (14,000, 18,100) *	11,100 (7880, 14,100) *	2350 (1870, 4440) *	n.s.				
17-Hydroxypregnanolone, C	−	3.31 (2.12, 4.85)	15.8 (8.15, 19.1)	59.9 (24.1, 124)	14.9 (13.6, 24.7)	H > S, L > S, K > S, L > H	<0.001	<0.001	0.025	0.091
[fmol/g]	+	4360 (4180, 5030) *	850 (628, 1570) *	18,100 (14,500, 25,000) *	11,200 (8250, 13,000) *	n.s.				
5β,20α-Tetrahydro-progesterone [fmol/g]	−	150 (127, 199)	983 (848, 1270)	35.8 (25.4, 58.9)	15.5 (12.5, 16.2)	H > S, L < S, K < S, L < H, K < H, K < L	<0.001	<0.001	<0.001	0.111
	+	447 (386, 497) *	794 (681, 896)	87.4 (65.7, 154) *	111 (81.2, 277) *	L < S, K < S, L < H, K < H				
5β,20α-Tetrahydro-progesterone, C [fmol/g]	−	39.9 (33.5, 48)	395 (324, 606)	89.1 (68.6, 134)	53.1 (41.3, 73.6)	H > S, L > S, K > S, L < H, K < H, K < L	<0.001	<0.001	0.002	0.006
	+	83.9 (80.1, 111) *	580 (448, 745)	214 (149, 216) *	229 (186, 265) *	H > S, K > S, L < H, K < H				
5β-Pregnane-3α,20α-diol	−	4.49 (3.92, 6.76)	2.39 (1.07, 3.16)	14.4 (4.96, 24.7)	0.159 (0.0398, 0.193)	H < S, L > S, K < S, L > H, K < H, K < L	<0.001	<0.001	<0.001	0.019
[pmol/g]	+	61.3 (57.3, 65.2) *	21.4 (17.4, 22.2) *	16.1 (14.4, 27.5)	8.98 (5.26, 13.4) *	H < S, L < S, K < S				
5β-Pregnane-3α,20α-diol, C	−	0.212 (0.125, 0.298)	0.12 (0.0511, 0.205)	0.148 (0.0316, 0.52)	0.189 (0.094, 0.271)	n.s.	<0.001	0.137	0.674	0.738
[pmol/g]	+	15.3 (13.6, 17.1) *	2.57 (2.16, 3.82) *	19 (13.4, 21.2) *	15.5 (10.2, 18.8) *	n.s.				
5β-Pregnane-3α,17,20α-triol	−	7.52 (4.89, 9.44)	48 (32.2, 117)	2.38 (1.99, 4.89)	1.42 (1.23, 2.04)	H > S, L < S, K < S, L < H, K < H, K < L	<0.001	<0.001	<0.001	0.001
[fmol/g]	+	220 (187, 317) *	487 (344, 557)	175 (155, 233) *	80.6 (55.5, 110) *	K < H				
Etiocholanolone	−	27 (19.8, 36.1)	215 (88.8, 323)	19.3 (15.9, 38.5)	8.52 (6.15, 9.95)	H > S, K < S, L < H, K < H, K < L	<0.001	<0.001	0.008	0.07
[fmol/g]	+	453 (412, 500) *	512 (379, 716) *	171 (141, 285) *	69.4 (49.8, 93.3) *	K < S, K < H				
Etiocholanolone, C	−	238 (205, 270)	31.2 (21.5, 40.7)	305 (244, 380)	155 (127, 186)	H < S, L > S, K < S, L > H, K > H, K < L	<0.001	<0.001	<0.001	0.942
[fmol/g]	+	883 (870, 962) *	106 (57.1, 143) *	611 (580, 650) *	578 (499, 665) *	H < S, K < S, L > H, K > H				

Note: Data are shown as median with quartiles; retransformed means in full cohort (Table 2) may differ due to sample size and statistical measure employed. Evaluated by mixed-design ANOVA (within-subject factors: PG, Biological Material (BM); random factor: Subject and PG × BM interaction). Post hoc pairwise comparisons between biological materials were performed separately for PG+ and PG− groups using Bonferroni correction (corrected α = 0.0083 for 6 comparisons per group). Multi-tissue analysis was performed on a representative subset with complete collection of all biological materials (n = 6 PG+, n = 21 PG−), optimized for detecting large effect sizes in peripheral metabolism. * *p* < 0.05 indicates significant difference vs. PG− rats.

**Table 4:** Percentage composition of the total 5β-steroid pool in the right hippocampus of PG+ rats (n = 16) and PG− rats (n = 27); data are presented as means ± SEM.

Steroid	Percentage of the Total 5β-Steroids	
	PG+	PG−
Pregnanolone (3α,5β-tetrahydroprogesterone; 3α,5β-THP)	44.98 ± 6.28% ***	5.42 ± 0.71%
Conjugated pregnanolone (3α,5β-THPC)	26.19 ± 2.89% ***	1.69 ± 0.44%
Conjugated epipregnanolone (3β,5β-tetrahydroprogesterone; 3β,5β-THPC)	0.15 ± 0.06% ***	1.15 ± 0.25%
Conjugated 5β,20α-tetrahydroprogesterone (3α,5β,20α-THPC)	0.22 ± 0.04% ***	4.85 ± 0.62%
5β-Pregnane-3α,20α-diol (3α,5β,20α-PD)	8.24 ± 1.11% **	22.96 ± 3.49%
Conjugated 5β-pregnane-3α,20α-diol (3α,5β,20α-PDC)	4.8 ± 0.86% **	2.35 ± 0.46%
5β-Pregnane-3β,20α-diol (3β,5β,20α-PD)	1.69 ± 0.26% ***	34.71 ± 4.52%
Conjugated 5β-pregnane-3β,20α-diol (3β,5β,20α-PDC)	0.77 ± 0.12% ***	12.71 ± 1.78%
17-Hydroxypregnanolone (5β-pregnane-3α,17-diol; 3α,5β,17-PD)	7.15 ± 0.89% ***	0.98 ± 0.32%
Conjugated 17-hydroxypregnanolone (3α,5β,17-PDC)	4.1 ± 0.88% ***	0.18 ± 0.02%
5α-Pregnane-3α,17,20α-triol (3α,5β,17,20α-THP)	0.31 ± 0.05% *	1.03 ± 0.4%
Etiocholanolone (3α-hydroxy-5β-androstane-17-one; 3α,5β-THA)	0.38 ± 0.09% ***	3.66 ± 0.92%
Conjugated etiocholanolone (3α,5β-THAC)	0.38 ± 0.12% *	1.84 ± 0.6%
Epietiocholanolone (3β-hydroxy-5β-androstane-17-one; 3β,5β-THA)	0.13 ± 0.05% ***	2.85 ± 0.51%
Conjugated epietiocholanolone (3β,5β-THAC)	0.17 ± 0.05% ***	1.49 ± 0.32%
Conjugated 5β-androstane-3α,17β-diol (3α,5β,17β-ADC)	0.33 ± 0.14% ***	2.13 ± 0.36%
Σ(5β-Steroids)	100 ± 7.17%	100 ± 7.84%

Differences tested by Mann–Whitney robust test * *p* < 0.05, ** *p* < 0.01, *** *p* < 0.001; THP = tetrahydroprogesterone; PD = pregnanediol, C (last letter in abbreviation) = conjugated steroid.

**Table 5:** Concentrations of 5α-reduced steroids in the right hippocampus (RH) of PG+ rats compared to PG− rats.

Steroid	Unit	Pregnanolone Glutamate (PG)		Sex		ANOVA (*p*-Value)		
						PG		
		PG+	PG−	Female	Male	*p*-Value	q (FDR)	Sex
5α-Dihydroprogesterone	pmol/g	1.17 (0.949, 1.45)	1.46 (1.24, 1.72)	1.61 (1.34, 1.94)	1.06 (0.878, 1.28) *	0.253	0.506	0.026
Allopregnanolone	pmol/g	2.96 (2.15, 3.99)	3.69 (2.93, 4.61)	4.37 (3.39, 5.56)	2.47 (1.84, 3.25) *	0.411	0.548	0.033
Allopregnanolone, C	pmol/g	1.35 (1.09, 1.65) **	0.739 (0.618, 0.88)	1.09 (0.899, 1.31)	0.927 (0.76, 1.12)	0.003	0.030	0.396
Isopregnanolone	pmol/g	2.83 (2.4, 3.38)	2.75 (2.42, 3.15)	2.69 (2.33, 3.13)	2.89 (2.49, 3.39)	0.853	0.853	0.622
Isopregnanolone, C	pmol/g	2.36 (2.03, 2.77)	1.84 (1.66, 2.05)	1.91 (1.7, 2.17)	2.25 (1.98, 2.59)	0.059	0.148	0.195
5α,20α-Tetrahydroprogesterone	pmol/g	9.79 (7.4, 12.9)	13.5 (10.9, 16.7)	12.7 (10, 16.2)	10.4 (8.09, 13.2)	0.198	0.453	0.389
5α,20α-tetrahydroprogesterone, C	pmol/g	15.8 (12.7, 19.3)	18 (15.4, 20.8)	18.7 (15.7, 21.9)	15.2 (12.5, 18.2)	0.471	0.548	0.243
5α-Pregnane-3α,20α-diol	pmol/g	18.5 (15, 23)	22 (18.5, 26.3)	23.1 (19, 28.3)	17.6 (14.7, 21.4)	0.381	0.548	0.164
5α-Pregnane-3α,20α-diol, C	pmol/g	21.1 (17.4, 25.7) *	14.2 (12.3, 16.4)	16.1 (13.7, 19.1)	18.4 (15.6, 21.9)	0.024	0.072 †	0.422
5α-Pregnane-3β,20α-diol	fmol/g	271 (149, 467) *	716 (485, 1040)	633 (403, 965)	312 (187, 502)	0.044	0.110 †	0.125
5α-Pregnane-3β,20α-diol, C	fmol/g	948 (727, 1210) *	542 (430, 671)	656 (514, 822)	802 (626, 1010)	0.025	0.072 †	0.383
17-Hydroxyallopregnanolone	pmol/g	2.73 (2.13, 3.47)	4.1 (3.42, 4.89)	4.44 (3.62, 5.4)	2.5 (2, 3.11) **	0.061	0.139	0.008
5α-Pregnane-3α,17,20α-triol	fmol/g	165 (122, 218)	257 (208, 315)	236 (186, 295)	182 (139, 234)	0.077	0.154	0.283
Androsterone	pmol/g	2.07 (1.73, 2.51)	2.42 (2.1, 2.82)	2.53 (2.14, 3.01)	2 (1.7, 2.36)	0.357	0.548	0.151
Androsterone, C	pmol/g	0.826 (0.731, 0.927) ***	0.458 (0.403, 0.517)	0.566 (0.496, 0.64)	0.695 (0.617, 0.777)	<0.001	<0.001	0.088
Epiandrosterone	fmol/g	221 (157, 296) *	386 (318, 461)	336 (265, 416)	261 (199, 333)	0.027	0.072 †	0.287
Epiandrosterone, C	fmol/g	292 (163, 511) *	94.9 (58.4, 152)	107 (61.8, 180)	262 (155, 432)	0.039	0.104 †	0.085
5α-Androstane-3α,17β-diol	fmol/g	207 (158, 276)	299 (239, 378)	207 (163, 265)	300 (231, 393)	0.158	0.395	0.141
5α-Androstane-3α,17β-diol, C	fmol/g	322 (273, 373)	253 (218, 290)	267 (227, 309)	307 (264, 352)	0.117	0.312	0.341
5α-Androstane-3β,17β-diol	fmol/g	227 (154, 331)	149 (108, 202)	190 (134, 266)	179 (125, 252)	0.231	0.495	0.858
5α-Androstane-3β,17β-diol, C	fmol/g	369 (196, 681) *	89.5 (52.6, 150)	197 (111, 344)	173 (95.8, 305)	0.018	0.063 †	0.811
11β-Hydroxyandrosterone, C	fmol/g	73.8 (46.6, 119) *	31.1 (22.3, 43.9)	58.7 (39.5, 88.2)	38.7 (26.2, 57.9)	0.037	0.104 †	0.290
11β-Hydroxyepiandrosterone, C	fmol/g	42.4 (24.5, 78.2) **	12.3 (8.8, 17.7)	19.5 (12.9, 30.7)	24.9 (16, 40.4)	0.009	0.045	0.578

Note: Data are presented as retransformed means with 95% confidence intervals. Relationships were evaluated by two-way ANOVA (factors: PG status and Sex). PG (1 mg/kg) was administered i.p. 5 min after intrahippocampal ET-1 injection; samples were collected 60 min post-injection. n = 16 (PG+), n = 27 (PG− rats). C = conjugated steroid. * *p* < 0.05, ** *p* < 0.01, *** *p* < 0.001 (nominal). Benjamini–Hochberg FDR-adjusted q-values are reported in the “q (FDR)” column. † q ≥ 0.05: exploratory finding requiring independent confirmation.

**Table 6:** Quantitative distribution of 5α-steroids across serum, hippocampus, liver, and kidney in PG+ rats versus PG− rats.

Steroid	PG	Biological Material (BM)				Significant Differences	ANOVA (*p*-Value)			
		Serum (S)	Hippocampus, Right (H)	Liver (L)	Kidney (K)	(*p* < 0.05), Bonferroni Multiple Comparisons				
							PG	BM	PG × BM	Subj(PG)
5α-Dihydroprogesterone	−	0.796 (0.607, 1.68)	1.82 (1.21, 2.38)	0.514 (0.289, 1.39)	1.09 (0.541, 2.04)	H > S, L < H, K < H	0.458	<0.001	0.173	0.005
[pmol/g]	+	1.26 (0.76, 1.58)	1.89 (1.66, 2.07)	0.312 (0.234, 1.34)	1.8 (1.62, 3.12) *	L < H				
Allopregnanolone	−	4.17 (3.38, 7.36)	4.74 (3.01, 9.29)	8.32 (4.28, 13.3)	9.86 (5.79, 12.1)	L > S, K > S, L > H, K > H	0.333	<0.001	0.155	<0.001
[pmol/g]	+	8.16 (6.32, 10.1) *	5.78 (3.66, 7.62)	7.42 (4.81, 9.41)	19.7 (11.5, 26.9) *	K > H				
Allopregnanolone, C	−	1.75 (1.3, 2.3)	0.765 (0.53, 1.75)	19 (12.8, 23.3)	8.17 (4.35, 10)	H < S, L > S, K > S, L > H, K > H, K < L	0.151	<0.001	0.814	0.010
[pmol/g]	+	1.83 (1.69, 2.51)	1.27 (1.12, 1.33)	18.3 (16.5, 20.8)	15.3 (6.67, 22.2)	L > S, K > S, L > H, K > H, K < L				
Isopregnanolone	−	0.178 (0.118, 0.275)	2.81 (1.95, 3.59)	0.858 (0.47, 1.53)	3.93 (2.74, 5.66)	H > S, L > S, K > S, L < H, K > H, K > L	0.439	<0.001	0.524	0.001
[pmol/g]	+	0.191 (0.0846, 0.267)	2.36 (1.47, 2.95)	0.492 (0.303, 1.1)	6.07 (3.44, 8.82)	H > S, L > S, K > S, L < H, K > H, K > L				
Isopregnanolone, C	−	1.31 (1.17, 1.99)	1.61 (1.41, 1.89)	8.3 (7.31, 11.6)	9.05 (7.41, 11.5)	L > S, K > S, L > H, K > H	0.982	<0.001	0.187	0.022
[pmol/g]	+	1.54 (1.48, 1.73)	1.56 (1.34, 1.83)	6.62 (6.41, 7.43)	11.6 (6.21, 17.9)	L > S, K > S, L > H, K > H				
17-Hydroxy-allopregnanolone [pmol/g]	−	5.56 (4.42, 6.95)	5.4 (3.31, 6.96)	10 (7.54, 18.7)	0.631 (0.558, 1.02)	L > S, K < S, L > H, K < H, K < L	0.960	<0.001	0.548	<0.001
	+	6.19 (5.22, 6.88)	3.93 (2.65, 5.34)	16 (9.79, 22.8)	0.927 (0.633, 1.01)	L > S, K < S, L > H, K < H, K < L				
17-Hydroxy-allopregnanolone, C [pmol/g]	−	5.69 (5.21, 6.89)	0.104 (0.0612, 0.191)	24.2 (16.9, 28.9)	13.6 (11.2, 18.1)	H < S, L > S, K > S, L > H, K > H, K < L	0.445	<0.001	0.450	0.008
	+	6.55 (5.11, 7.12)	0.118 (0.0587, 0.153)	21.8 (17.3, 26.6)	12 (10.6, 15.2)	H < S, L > S, K > S, L > H, K > H, K < L				
5α,20α-Tetrahydro-progesterone [pmol/g]	−	38.6 (22.2, 63)	15.2 (14.1, 25.3)	6.49 (4.68, 10.2)	7.06 (5.26, 10.3)	H < S, L < S, K < S, L < H, K < H	0.509	<0.001	0.221	<0.001
	+	47.4 (41.3, 66) *	17.5 (14, 18.6) *	5.08 (3.26, 18.7)	10 (7.59, 28.2) *	H < S, L < S, K < S, L < H				
5α,20α-Tetrahydro-progesterone, C [pmol/g]	−	7.91 (6.04, 11.3)	20.3 (15.1, 28.3)	6.82 (4.53, 9.5)	7.7 (4.97, 11.4)	H > S, L < H, K < H	0.176	<0.001	0.788	<0.001
	+	9.16 (6.81, 14.6)	22.5 (21.2, 26.5)	8.15 (7.22, 8.82)	6.77 (5.95, 11.8) *	H > S, L < H, K < H				
5α-Pregnane-3α,20α-diol	−	454 (303, 742)	23.4 (17.4, 43.7)	19.5 (10.5, 34.8)	28.5 (18.5, 43.5)	H < S, L < S, K < S	0.783	<0.001	0.889	<0.001
[pmol/g]	+	597 (465, 672)	25.4 (19.1, 38.8)	24.5 (16.6, 34.9)	39.4 (21.4, 54.4)	H < S, L < S, K < S				
5α-Pregnane-3α,20α-diol, C	−	145 (105, 191)	15.4 (11.5, 19.3)	221 (139, 314)	55.6 (49, 65)	H < S, L > S, K < S, L > H, K > H, K < L	0.844	<0.001	0.123	<0.001
[pmol/g]	+	145 (143, 165)	14.4 (11.4, 21.1)	167 (154, 185) *	57.7 (44.9, 74.3)	H < S, K < S, L > H, K > H, K < L				
5α-Pregnane-3β,20α-diol	−	4.41 (2.18, 6.5)	1.27 (0.617, 1.65)	0.736 (0.66, 1.06)	2.97 (1.4, 5.13)	H < S, L < S, K > H, K > L	0.424	<0.001	0.098	<0.001
[pmol/g]	+	4.5 (3.17, 6.13)	0.738 (0.57, 1.46)	0.885 (0.652, 1.7)	4.74 (3.14, 11) *	H < S, L < S, K > H, K > L				
5α-Pregnane-3β,20α-diol, C	−	6.35 (5.12, 7.27)	0.468 (0.212, 0.658)	12.8 (10.1, 16.1)	12.9 (7.41, 14)	H < S, L > S, K > S, L > H, K > H	0.083	<0.001	<0.001	0.002
[pmol/g]	+	6.7 (6.14, 7.15)	0.446 (0.321, 0.562)	10.6 (9.53, 11.4)	18.5 (11.7, 25.6) *	H < S, K > S, L > H, K > H, K > L				
5α-Pregnane-3α,17,20α-triol	−	210 (160, 228)	289 (184, 400)	204 (53.6, 595)	19.2 (17.8, 26)	K < S, K < H, K < L	0.983	<0.001	0.094	0.005
[fmol/g]	+	165 (154, 228)	193 (145, 244)	348 (219, 486)	26.6 (19.7, 29.8)	L > S, K < S, L > H, K < H, K < L				
Androsterone	−	1.85 (1.43, 2.33)	2.79 (2.16, 3.96)	1.88 (1.41, 2.84)	0.43 (0.348, 0.619)	H > S, K < S, K < H, K < L	0.594	<0.001	0.651	0.002
[pmol/g]	+	2.37 (2.03, 2.55)	2.14 (1.76, 3.57)	2.48 (1.5, 4.44)	0.5 (0.459, 1.03)	K < S, K < H, K < L				
Androsterone, C	−	20.6 (19, 27.4)	0.414 (0.333, 0.514)	72 (50, 98.3)	30.5 (27, 47.1)	H < S, L > S, K > S, L > H, K > H, K < L	0.217	<0.001	0.217	0.034
[pmol/g]	+	20.2 (18.6, 25)	0.545 (0.38, 0.887)	54.6 (53.4, 59.5) *	30.9 (27.2, 33.2)	H < S, L > S, L > H, K > H, K < L				
Epiandrosterone, C	−	0.563 (0.477, 0.712)	0.0565 (0.0195, 0.0865)	1.04 (0.769, 1.37)	1.45 (1.08, 1.96)	H < S, L > S, K > S, L > H, K > H, K > L	0.227	<0.001	0.058	0.385
[fmol/g]	+	0.669 (0.517, 0.771)	0.0449 (0.014, 0.104)	0.7 (0.672, 0.782) *	1.19 (0.988, 1.63)	H < S, K > S, L > H, K > H, K > L				
5α-Androstane-3α,17β-diol	−	0.247 (0.136, 0.473)	0.368 (0.151, 0.513)	0.424 (0.246, 0.784)	0.125 (0.104, 0.192)	L > S, K < S, K < H, K < L	0.603	<0.001	0.311	<0.001
[pmol/g]	+	0.187 (0.137, 0.219)	0.233 (0.199, 0.38)	0.857 (0.648, 1.28) *	0.135 (0.127, 0.246)	L > S, L > H, K < L				
5α-Androstane-3α,17β-diol, C [pmol/g]	−	1.92 (1.47, 2.72)	0.227 (0.151, 0.299)	53.1 (39.9, 64.7)	5 (4.17, 6.37)	H < S, L > S, K > S, L > H, K > H, K < L	0.414	<0.001	0.790	<0.001
	+	1.83 (1.7, 2.09)	0.201 (0.115, 0.293)	42.6 (38, 46.7)	4.65 (4.3, 5.06)	H < S, L > S, K > S, L > H, K > H, K < L				
5α-Androstane-3β,17β-diol	−	14.4 (8.97, 32.7)	93.6 (54.1, 202)	22 (17.4, 48.3)	8.86 (7.36, 11.5)	H > S, L > S, L < H, K < H, K < L	0.391	<0.001	0.089	0.214
[fmol/g]	+	16.5 (5.02, 21.4)	201 (140, 311)	31.8 (19.3, 39.5)	13.9 (12.8, 17.5)	H > S, L < H, K < H				
5α-Androstane-3β,17β-diol, C [fmol/g]	−	26 (16.4, 44)	34.4 (17, 85)	503 (409, 703)	145 (109, 188)	L > S, K > S, L > H, K > H, K < L	0.373	<0.001	0.906	0.504
	+	20.8 (12.2, 53.9)	34 (22.8, 60.6)	381 (330, 453)	129 (93.3, 223)	L > S, K > S, L > H, K > H, K < L				
11β-Hydroxyandrosterone	−	945 (556, 1050)	451 (170, 600)	2320 (970, 2850)	36.9 (30.4, 52.7)	H < S, L > S, K < S, L > H, K < H, K < L	0.641	<0.001	0.501	0.022
[fmol/g]	+	710 (623, 853)	365 (128, 548)	2190 (1720, 2710)	53.1 (47.2, 64.1)	H < S, L > S, K < S, L > H, K < H, K < L				
11β-Hydroxyandrosterone, C [fmol/g]	−	1120 (683, 1560)	36.4 (23.5, 53.2)	1520 (1190, 1650)	2890 (1650, 3600)	H < S, K > S, L > H, K > H, K > L	0.308	<0.001	0.652	0.019
	+	888 (623, 1240)	69.4 (55.3, 86.1)	1470 (1100, 1560)	1760 (1370, 2620)	H < S, K > S, L > H, K > H				
11β-Hydroxyepiandrosterone [fmol/g]	−	111 (56.7, 152)	67.7 (34.3, 135)	178 (111, 255)	21.4 (15.3, 34.8)	H < S, K < S, L > H, K < H, K < L	0.103	<0.001	0.989	0.002
	+	142 (97.8, 176)	97.5 (43.2, 176)	251 (158, 370)	32.3 (22.3, 41.4)	K < S, L > H, K < L				
11β-Hydroxyepiandrosterone, C [fmol/g]	−	779 (603, 1040)	9.1 (5.73, 15.6)	1420 (1120, 1750)	1970 (1510, 2460)	H < S, L > S, K > S, L > H, K > H, K > L	0.590	<0.001	0.702	<0.001
	+	1010 (716, 1290)	14.9 (6.55, 60.2)	1490 (1070, 1620)	1870 (1640, 2750)	H < S, K > S, L > H, K > H, K > L				

Note: Data are shown as median with quartiles. Evaluated by mixed-design ANOVA (within-subject factors: PG, Biological Material (BM); random factor: Subject and PG × BM interaction). Post hoc pairwise comparisons between biological materials were performed separately for PG+ and PG− groups using Bonferroni correction (corrected α = 0.0083 for 6 comparisons per group). * *p* < 0.05 indicates significant difference vs. PG− rats. Multi-tissue analysis was performed on a representative subset with complete collection of all biological materials (n = 6 PG+, n = 21 PG−), optimized for detecting large effect sizes in peripheral metabolism. * *p* < 0.05 indicates significant difference vs. PG− rats.

### 2.2. Correlations Among Steroids in Serum and Hippocampus

Pearson’s correlation analysis was utilized to evaluate the linear relationships among individual steroids within serum and the hippocampus, as well as the coupling between these two biological compartments.

#### 2.2.1. Correlations Within the 5β-Steroid Pathway

The administration of PG resulted in distinct correlation patterns among 5β-reduced steroids compared to the PG− rats.

Serum and hippocampal correlations (PG+): In the serum of PG+ rats (Table S1), strong positive correlations were observed between the parent compound, pregnanolone, and its conjugated form (r = 0.8), as well as with epipregnanolone (r = 0.8). Similarly, within the hippocampus (Table S2), pregnanolone showed significant positive correlations with downstream metabolites, including 5β,20α-tetrahydroprogesterone (r = 0.8) and 5β-pregnane-3α,20α-diol (r = 0.9). Conversely, the correlation profile in the PG− hippocampus (Table S3) provided distinct mechanistic insights. Most notably, the coupling between pregnanolone and its conjugate was entirely absent (r = 0.0), contrasting sharply with the serum profile (Table S4). However, significant positive correlations persisted between downstream reduced metabolites (e.g., 5β,20α-tetrahydroprogesterone and 5β-pregnane-3α,20α-diol, r = 0.7). In contrast, the analysis of serum correlations in the control group (Table S4) reflects the physiological baseline of steroidogenic regulation. While the correlation network was less dense compared to the pharmacologically saturated state, a strong positive relationship was maintained between endogenous pregnanolone and its conjugate (r = 0.7), as well as with its direct metabolite 5β-pregnane-3α,20α-diol (r = 0.7). However, unlike the PG+ group, significant correlations with downstream androgen metabolites (e.g., etiocholanolone) were absent.

Serum-hippocampus coupling (PG+ vs. PG− rats): A marked difference was observed when comparing serum and the right hippocampus. In PG+ animals (Table S5), the hippocampal levels of 5β-steroids were strongly correlated with their serum concentrations. Specifically, serum pregnanolone positively correlated with hippocampal pregnanolone (r = 0.7) and hippocampal epipregnanolone (r = 0.9). Furthermore, the serum pregnanolone conjugate showed a strong positive correlation with hippocampal pregnanolone conjugate (r = 0.8).

In contrast, the PG− group (Table S6) exhibited generally weaker or non-significant relationships between peripheral and central compartments. While serum pregnanolone showed a moderate correlation with hippocampal pregnanolone (r = 0.6), correlations between serum pregnanolone and other hippocampal metabolites (e.g., epipregnanolone, r = −0.1) were not statistically significant.

#### 2.2.2. Correlations Within the 5α-Steroid Pathway

The analysis also evaluated the relationships among endogenous 5α-reduced neurosteroids, which are not metabolic products of the administered drug.

Correlations in PG+ rats: In the PG+ group, significant positive correlations were detected within the 5α-pathway. In the serum (Table S7), conjugated allopregnanolone correlated strongly with conjugated isopregnanolone (r = 1.0) and conjugated 5α-pregnane-3α,20α-diol (r = 0.8). Within the hippocampus (Table S8), free allopregnanolone showed a positive correlation with 5α-dihydroprogesterone (r = 0.6) and a strong correlation with 5α-pregnane-3α,20α-diol (r = 0.9). In the PG− group, the analysis of serum 5α-steroids (Table S9) revealed a remarkably highly interconnected metabolic network, contrasting with the sparser correlations observed in the 5β-pathway (Table S4). Endogenous allopregnanolone exhibited robust correlations not only with its conjugate (r = 0.8) and precursor 5α-dihydroprogesterone (r = 0.7), but also with downstream metabolites such as androsterone (r = 0.7). Finally, the profile of the PG− hippocampus (Table S10) offered an interesting contrast to the 5β-pathway. Unlike the complete decoupling observed for pregnanolone, endogenous allopregnanolone maintained a moderate positive correlation with its conjugate (r = 0.6). This suggests a tighter physiological equilibrium between peripheral supply and central levels for the dominant endogenous neurosteroid. Crucially, however, the autonomy of local synthesis was confirmed by the strong correlation between the precursor 5α-dihydroprogesterone and allopregnanolone (r = 0.7), demonstrating that the hippocampus actively synthesizes 5α-neuroactive steroids alongside their uptake from circulation.

Serum-hippocampus coupling (5α-steroids): Additionally, the serum allopregnanolone conjugate correlated with the hippocampal allopregnanolone conjugate (r = 0.5) and showed a strong correlation with the hippocampal 11β-hydroxyandrosterone conjugate (r = 0.9). In contrast, the serum-hippocampus correlation for androsterone conjugate was markedly weaker (r = 0.1, Table S11) compared to the pregnanolone conjugate (r = 0.8, Table S5), suggesting differential blood–brain barrier permeability for androgenic versus progestogenic steroid conjugates, possibly due to selective substrate affinity for organic anion transporters. These significant cross-compartment correlations in the PG+ group contrast with the variable correlation patterns observed in the PG− group (Table S12), where, for example, the relationship between serum and hippocampal allopregnanolone was also positive (r = 0.8), but other metabolite pairings showed differing degrees of association. All correlations with r ≥ 0.5 reported in this section were statistically significant (*p* < 0.05, Spearman’s rank correlation).

### 2.3. OPLS Multivariate Regression Analysis of Steroidome Relationships

#### 2.3.1. Serum–Hippocampus Coupling: Loss of Central Autonomy Under Pharmacological Saturation

To characterize the functional coupling between the periphery and the central compartment, OPLS models were constructed to predict hippocampal (RH) steroid levels based on the serum steroidome (Table 7). This analysis was performed on the multi-tissue subset (n = 6 PG+, n = 21 PG−), where complete serum and tissue profiles enabled robust multivariate modeling of peripheral-to-central steroid transport. In PG+ animals, PG administration markedly strengthened the peripheral-to-brain coupling within the 5β-steroid axis. Serum-to-RH models yielded high predictive values (typically R > 0.9), indicating that under pharmacological saturation, the hippocampal steroid profile becomes a direct imprint of the systemic biotransformation. Specifically, RH pregnanolone levels (R = 0.90 **) were predicted by a broad set of serum markers, including the parent compound, its conjugates, and downstream androgenic metabolites (etiocholanolone, epietiocholanolone). Crucially, the model for conjugated pregnanolone was significant and robust only in PG+ rats (R = 0.77 **), whereas in control animals (PG−), no significant relationship between the serum and hippocampal conjugate pools was found. In contrast, PG− rats exhibited a distinct “metabolic autonomy”. The predictive power of serum models was significantly weaker (R ≈ 0.59 for pregnanolone) and relied on a narrower set of direct precursors. This confirms that under physiological conditions, the blood–brain barrier (BBB) maintains central levels relatively independent of peripheral fluctuations.

#### 2.3.2. Intra-Hippocampal Relationships: Modular Segregation of Free and Conjugated Pools

To analyze the internal metabolic architecture of the hippocampus, separate OPLS models were generated for each 5β-steroid (Table 8). The analysis revealed a striking modular organization of the central steroidome, particularly in the PG+ group. The data demonstrated a fundamental segregation between two pharmacological compartments:

The Unconjugated (Free) Axis: Free pregnanolone showed robust positive associations with its direct reduction products (5β,20α-tetrahydroprogesterone and 5β-pregnane-3α,20α-diol). This cluster confirms the continuity of the local enzymatic processing (AKR activity) of lipophilic steroids.

The Conjugated Module: Conjugated steroids formed a separate, highly interconnected cluster (e.g., conjugated pregnanolone positively predicted conjugated 17-hydroxypregnanolone and etiocholanolone) that frequently exhibited significant inverse associations with the free steroid axis.

For instance, in PG+ rats, conjugated pregnanolone negatively predicted the levels of free active metabolites. Furthermore, downstream androgenic end-products (etiocholanolone and epietiocholanolone) strictly followed this segregation: their free forms aligned with the free axis, while their conjugated forms aligned with the conjugated module. This statistical decoupling suggests that these two pools do not rapidly equilibrate within the tissue.

## 3. Discussion

This study provides the first comprehensive pharmacokinetic analysis of pregnanolone glutamate (PG) and its metabolites in postnatal rats subjected to ET-1-induced focal hippocampal ischemia (Figure 1). Our results demonstrate that systemically administered PG undergoes rapid peripheral metabolism, primarily via deconjugation to pregnanolone (P) followed by further biotransformation including 17-hydroxylation and sulfation, resulting in substantial serum concentrations of sulfated 5β-steroids (S5β). Notably, these polar sulfated metabolites were detected in the ischemic hippocampus alongside P and its 5α-reduced derivatives, indicating BBB penetration of both unconjugated and conjugated neurosteroids. Importantly, all hippocampal measurements were obtained from ischemic tissue, which has implications for the interpretation of BBB permeability and metabolic profiles, as discussed below.

### 3.1. Pregnanolone Glutamate as a Peripheral Shuttle: The “Parallel Influx” Mechanism

The pharmacokinetic profile of pregnanolone glutamate (PG) revealed a sophisticated dual mechanism of action, which we define as a “parallel influx”. By combining the rapid hydrolysis of a prodrug with the carrier-mediated delivery of the intact molecule, PG overcomes the classic limitations of neurosteroid therapeutics.

First, the steep concentration gradient of free pregnanolone observed at 60 min post-administration (Serum > Liver > Kidney > Hippocampus; Table 3) is consistent with enzymatic cleavage by plasma and hepatic esterases, primarily via γ-glutamyl hydrolase (GGH) [17], although the kinetics of this process cannot be fully resolved from a single time-point. This releases lipophilic pregnanolone, generating a robust systemic pool that drives passive diffusion across the blood–brain barrier (BBB).

It should be noted that transcardial perfusion was not performed in this study; however, quantitative estimation using published cerebral blood volume data [18] indicates that residual intravascular blood can account for at most 19–29% of the measured hippocampal pregnanolone concentration (see Section 4), with the majority of the signal (71–81%) attributable to tissue-resident steroid. Moreover, the analyte-specific tissue gradients observed (e.g., hippocampal enrichment of 5β,20α-tetrahydroprogesterone relative to serum) are inconsistent with passive vascular carryover as the primary source of the measured brain levels.

Crucially, however, the OPLS multivariate analysis provides definitive statistical proof that PG is not merely a prodrug. The intra-hippocampal analysis (Section 2.3.2) uncovered a phenomenon of “metabolic stratification”, where free and conjugated steroid pools form two statistically independent modules within the tissue. If rapid central conjugation or hydrolysis were the dominant forces, these profiles would blend. Instead, their segregation implies that lipophilic metabolites enter via passive diffusion to feed the active free pool, while hydrophilic conjugates are imported via carrier-mediated transport into a “protected” reservoir.

This supports the intact transport hypothesis. Unlike endogenous sulfates, which are typically excluded from the adult brain [15,19,20], the glutamate conjugate utilizes a specific, high-capacity transport mechanism [17,21]. The strong correlation between serum and hippocampal conjugated pregnanolone levels in the PG+ group (Table 7) validates this carrier-mediated BBB translocation, likely involving organic anion transporting polypeptides (OATPs) or excitatory amino acid transporters (EAATs) [14,17,22,23,24,25]. Following brain entry, pregnanolone glutamate ensures immediate neurosteroid availability via enzymatic hydrolysis to pregnanolone, while providing sustained delivery of the intact precursor molecule [17], thereby combining rapid GABAergic modulation with prolonged neuroprotection.

### 3.2. The “Metabolic Sink” Hypothesis: Peripheral Origin of Metabolites in the Ischemic Hippocampus

A pivotal question regarding the pharmacodynamics of PG is whether the diverse spectrum of 5β-metabolites detected in the CNS originates from local neurosteroidogenesis or peripheral import. Our findings provide compelling evidence for the “metabolic sink” hypothesis [17], demonstrating that the brain passively accumulates metabolites primarily generated in systemic organs.

The OPLS analysis serves as the mechanistic cornerstone for this conclusion. We observed a dramatic transition from physiological autonomy to a transport-dominated state. In control animals, the brain regulates its steroid levels independently (weak serum-to-brain coupling). In contrast, the “saturated” brain in PG+ animals becomes a passive recipient, evidenced by extremely strong predictive models (R > 0.9) where hippocampal levels directly mirror the systemic biotransformation.

This statistical finding is biologically substantiated by the specific enzymatic signatures of the detected metabolites [17]. We observed a massive surge in hippocampal conjugated pregnanolone (rising nearly 500-fold). Given that the type 2A1 sulfotransferase (SULT2A1) is absent or negligible in the rodent brain [26,27], these conjugates cannot be synthesized locally and must originate from the periphery. Furthermore, the substantial accumulation of 17-hydroxypregnanolone and etiocholanolone unequivocally points to hepatic biotransformation [17], as the required enzyme CYP17A1 (steroid cytochrome P450 family 17 subfamily A member 1) is not expressed in neural tissue [28,29]. Inhibition of CYP17A1 has been shown to produce antidopaminergic effects in rodent models of sensorimotor gating [28], suggesting that 17-hydroxylated neurosteroids may modulate multiple neurotransmitter systems beyond their direct GABAergic actions [29]. The hippocampal concentration of 17-hydroxypregnanolone (16 pmol/g, Table 3) was comparable to serum levels (11.7 pmol/g), indicating efficient blood–brain barrier penetration of this peripherally generated metabolite. Sulfated neurosteroids can block extrasynaptic NMDA receptors [30], suggesting that peripherally generated conjugates may retain distinct pharmacological activity upon central accumulation.

Consequently, the presence of these compounds confirms their systemic origin. While the brain acts as a metabolic sink, the resulting neurosteroid profile remains distinct from plasma due to the “parallel influx” mechanism described above, which regulates the final central concentrations.

### 3.3. Metabolic Pathway Selectivity: Preserving Endogenous Neuroprotection

Safety and specificity are paramount for neuroactive steroid therapy. Our results demonstrate that PG administration induces a “metabolic segregation”, selectively saturating the pharmacological 5β-pathway while strictly preserving the homeostasis of the endogenous 5α-neuroprotective pathway within the brain.

While 5β-pregnanolone levels in the hippocampus surged nearly 170-fold (Table 2), causing a fundamental shift in the steroidomic profile toward the active parent compound, the hippocampal levels of allopregnanolone—the brain’s potent endogenous neuroprotectant—remained statistically unchanged (*p* = 0.411; Table 5). This stability is vital. Correlation analysis further highlights the resilience of the neuroprotective 5α-axis, which maintains tight homeostatic regulation independent of the exogenous 5β-steroid flood.

Finally, the strong correlation between the precursor 5α-dihydroprogesterone and allopregnanolone confirms that the hippocampus actively synthesizes 5α-neuroactive steroids locally, consistent with the demonstrated expression of key neurosteroidogenic enzymes (P450scc, 3β-HSD, 5α-reductase type I) in hippocampal pyramidal neurons [31]. This dual-source mechanism reinforces the resilience of the 5α-axis against external fluctuations, ensuring that the therapeutic administration of PG does not compromise the brain’s intrinsic neuroprotective capacity. Our findings are consistent with recent studies demonstrating the neuroprotective effects of allopregnanolone in neonatal hypoxic-ischemic models [32], supporting the therapeutic potential of pregnanolone glutamate in perinatal brain injury. The preserved endogenous 5α-neurosteroid pathway observed in our study aligns with evidence that allopregnanolone enhances endogenous neurogenesis and produces sustained neuroprotective actions following ischemic insults [33].

Sex-specific effects: Two-way ANOVA analysis revealed a significant main effect of sex for select 5α-steroids (allopregnanolone, *p* = 0.033; 17-hydroxyallopregnanolone, *p* = 0.008; 5α-dihydroprogesterone, *p* = 0.026; Table 5). However, the primary finding of preserved 5α-pathway homeostasis (unchanged free allopregnanolone, *p* = 0.411) held true for both sexes. For the major 5β-steroids induced by PG (pregnanolone, 17-hydroxypregnanolone, 5β-pregnane-3α,20α-diol), no significant sex × PG interaction was detected, indicating that the metabolic segregation mechanism is independent of sex. Future studies should employ larger sex-stratified cohorts to determine whether sex-specific pharmacokinetics influence therapeutic dosing in neonatal populations.

Notably, sex-specific differences in neurosteroid sensitivity have been demonstrated in adult models, where female mice required 4-fold lower doses of allopregnanolone for neuroprotection compared to males following cardiac arrest, correlating with greater potentiation of GABAergic inhibitory postsynaptic currents [34]. Interestingly, this sex difference was not observed in miniature IPSCs (action potential-independent events), suggesting that the enhanced sensitivity in females involves mechanisms beyond postsynaptic GABA_A_R binding, potentially including presynaptic modulation or calibration by endogenous neurosteroid levels [34,35]. While neonatal sex differences in neurosteroid pharmacology remain poorly characterized, and the immature brain may exhibit distinct steroidogenic enzyme expression compared to adults, the demonstrated sex differences in allopregnanolone sensitivity underscore the importance of evaluating pregnanolone glutamate efficacy in both male and female neonatal models. Given that pregnanolone—the primary metabolite of PG—shares structural and functional similarity with allopregnanolone as a GABA_A_R modulator, sex-stratified dosing strategies may be warranted for clinical translation, particularly in populations where endogenous neurosteroid tone differs between sexes or across developmental stages.

It is important to note that the present study did not directly measure neuroprotective endpoints such as lesion volume, neuronal cell counts, or behavioral outcomes. The term ‘neuroprotective’ as used here refers to the well-characterized pharmacological properties of the detected metabolites—particularly pregnanolone and allopregnanolone as GABA_A_R positive allosteric modulators—as established in prior electrophysiological, in vitro, and in vivo studies [16,17,36,37,38,39]. The preservation of the endogenous 5α-pathway observed herein is interpreted as pharmacokinetically favorable for neuroprotection based on these known activities, rather than as a direct demonstration of neuroprotective efficacy in the current experimental paradigm.

### 3.4. Functional Implications: Polypharmacology and Intracerebral Buffering

The pharmacokinetic profile of PG translates into a unique functional strategy. Rather than acting as a single agent, PG functions as a “molecular shuttle” that utilizes the body’s metabolic machinery to generate a synergistic “cocktail” of neuroactive metabolites (polypharmacology). As detailed in Table 2, the rapid hepatic conversion delivers not only the parent steroid but also biologically significant quantities of downstream metabolites like 17-hydroxypregnanolone and 5β-pregnane-3α,20α-diol. Since these compounds retain varying degrees of GABAergic activity, they likely contribute synergistically to the reported anticonvulsant effects [16,17,36].

Furthermore, our data reveal an intrinsic CNS safety mechanism: “intracerebral buffering”. The OPLS multivariate analysis demonstrated strong metabolic coupling within the hippocampus, with a highly predictive model for conjugated pregnanolone (R = 0.912; Table 8), indicating robust local steroid processing. The preferential hippocampal retention of 3β-isomers and oxidized metabolites suggests active local isomerization of the potent GABA-agonist pregnanolone (3α) to its isomer epipregnanolone (3β), which acts as a negative modulator.

The distinct accumulation of oxidized (3-oxo) and isomerized (3β) steroids in the hippocampus suggests that the brain actively utilizes oxidative enzymes—specifically 17β-hydroxysteroid dehydrogenases (HSD17B) such as HSD17B10—to dampen excessive GABAergic tone. Unlike the liver, where phase II metabolism clears these products, the brain retains these “regulatory” metabolites to fine-tune inhibitory tone. This active intracerebral conversion serves as a crucial buffer against oversedation. This observation is consistent with our recent study demonstrating a similar 3α-to-3β interconversion in the male rat brain, where allopregnanolone administration led to significant cerebral accumulation of its 3β-epimer [40]. Thus, PG offers a therapeutic advantage over the direct administration of labile free steroids, providing sustained neuroactive support while engaging local mechanisms that prevent excessive inhibition.

We note that while the core findings of this study—including the massive increases in pregnanolone, its conjugate, 17-hydroxypregnanolone, and 5β-pregnane-3α,20α-diol—withstand stringent FDR correction (q < 0.001; Table 2), a subset of nominally significant changes in minor downstream metabolites (e.g., epietiocholanolone conjugate, 5β-androstane-3α,17β-diol conjugate) should be interpreted as exploratory observations pending independent replication (Table 2, footnote). Importantly, the unchanged levels of free allopregnanolone (q = 0.548; Table 5)—the central finding supporting metabolic segregation—are robustly non-significant even without correction, reinforcing the conclusion that the endogenous 5α-neuroprotective pathway is preserved under PG treatment.

### 3.5. GABAergic Modulation as the Dominant Neuroprotective Pathway: Quantitative Implications of Peripheral Hydrolysis and Receptor-Specific Mechanisms

Our study establishes pregnanolone glutamate (PG) as a dual-mechanism neurotherapeutic where GABAergic modulation dominates the neuroprotective response. The majority of systemically administered PG undergoes rapid peripheral hydrolysis by esterases, primarily via γ-glutamyl hydrolase (GGH) [9], releasing free pregnanolone (P), which readily crosses the BBB due to its lipophilic nature [16,17,36]. This is evidenced by the steep serum-to-hippocampus gradient (404→107 pmol/g for free pregnanolone; Table 3), while direct penetration of the polar conjugate remains limited (42.3 pmol/g; Table 2).

The quantitative dominance of free pregnanolone over intact PG in the hippocampus (~2.5-fold higher concentration: 107 vs. 42.3 pmol/g; Table 3) indicates that the primary neuroprotective mechanism operates through GABA_A_R potentiation rather than direct NMDA receptor antagonism. This interpretation is further supported by the OPLS multivariate analysis (Table 7), which revealed strong serum-to-hippocampus coupling for free pregnanolone (R = 0.90 **) but weaker coupling for conjugated PG (R = 0.77 **), confirming that the lipophilic free steroid—not the polar conjugate—drives the central pharmacological response.

Notably, the hippocampal accumulation of 17-hydroxypregnanolone (16 pmol/g; Table 3) provides additional mechanistic insight into PG’s polypharmacological profile. This metabolite is generated via hepatic CYP17A1 activity [28,29] and subsequently crosses the blood–brain barrier, contributing to the neuroactive “cocktail” delivered by PG [29]. While CYP17A1 is absent from adult rodent brain tissue [28,29], inhibition of this enzyme has been shown to produce antipsychotic-like effects in models of dopaminergic hyperfunction [28,29], suggesting that 17-hydroxylated neurosteroids may modulate neurotransmitter systems beyond their direct GABAergic actions [29]. The combined action of pregnanolone and 17-hydroxypregnanolone as GABA_A receptor modulators likely accounts for the robust neuroprotective efficacy observed in our pFCI model [16,17,36].

Due to its polar glutamate moiety, PG exhibits limited BBB permeability and likely requires active transport mechanisms (e.g., OATPs, EAATs) for direct CNS entry [14,17,21,22,23,24]. This contrasts with sulfated neurosteroids such as pregnenolone sulfate, which modulate NMDA receptors through extracellularly directed sites [13] but exhibit even more restricted BBB penetration [14,15]. The glutamate conjugate strategy employed by PG thus represents a pharmacokinetic optimization over sulfated derivatives, combining improved CNS delivery with retained neuromodulatory activity. However, the modest hippocampal accumulation of conjugated PG (42.3 pmol/g) suggests that this transport pathway, while present, plays a secondary role compared to the massive influx of peripherally generated pregnanolone. In contrast, pregnanolone—generated peripherally via hydrolysis [17]—efficiently penetrates the brain and acts as a potent positive allosteric modulator of GABA_A_R, enhancing inhibitory neurotransmission and providing neuroprotection through multiple mechanisms, including a reduction in excitotoxicity, attenuation of neuroinflammation, and stabilization of mitochondrial function [37,38,39]. Notably, GABAergic neuroprotection has been identified as a promising experimental strategy specifically for perinatal hypoxic-ischemic encephalopathy [41].

While PG itself can modulate NMDA receptors when present in sufficient concentrations [4,13], the dominant mechanism underlying the therapeutic effects observed in our pFCI model is GABA_A_R-mediated, driven by pregnanolone and its metabolites (e.g., 17-hydroxypregnanolone) [17,36] rather than the intact PG molecule [29,42,43]. This interpretation is further supported by the pharmacological profile of allopregnanolone (the 5α-isomer, structurally analogous to pregnanolone), which exhibits similar GABA_A_R modulatory properties and neuroprotective efficacy [37,38,39]. Importantly, both progesterone and allopregnanolone have been shown to attenuate blood–brain barrier disruption following permanent focal ischemia by downregulating matrix metalloproteinases (MMP-9, MMP-2) and preserving tight junction proteins (occludin, claudin-5) [44], mechanisms that likely contribute to the preserved BBB integrity and reduced infarct volume observed in our study. The shared mechanism between these neurosteroids underscores the critical role of GABA_A_R potentiation in mediating protection against perinatal brain injury.

Critically, PG induces “metabolic segregation” within the CNS: the pharmacological 5β-pathway saturates (~170-fold pregnanolone increase; Table 2) while endogenous 5α-pathway (allopregnanolone) homeostasis remains preserved (*p* = 0.411; Table 5). This selective saturation is mechanistically significant, as it demonstrates that exogenous PG administration does not disrupt the brain’s intrinsic neuroprotective machinery. The OPLS intra-hippocampal analysis (Table 8) further revealed a modular organization of the central steroidome, with free and conjugated steroids forming two statistically independent compartments. Preferential hippocampal accumulation of 3β-isomers (e.g., epipregnanolone) suggests autonomous regulatory buffering via oxidative HSD17B enzymes [40], protecting against excessive GABAergic inhibition. This active intracerebral conversion serves as a crucial safety mechanism, preventing oversedation while maintaining therapeutic efficacy.

PG is not merely a prodrug or an active drug—it is a source of multiple neuroactive metabolites (Figure 1) with established GABAergic activity that were previously shown to synergistically enhance the positive outcome of perinatal focal cerebral ischemia in functional studies [16,17]. By utilizing the brain as a ‘metabolic sink’ for peripherally generated metabolites—including 17-hydroxypregnanolone, 5β-pregnane-3α,20α-diol, and etiocholanolone [17]—PG delivers a polypharmacological cocktail of metabolites with demonstrated GABAergic and neuroprotective properties [17,36,37,38,39] without disrupting endogenous neurosteroid homeostasis. We emphasize that the present study establishes the pharmacokinetic basis for this polypharmacological delivery; the direct correlation between the specific tissue concentrations reported here and the magnitude of neuroprotective outcomes remains to be established in future correlative studies. This unique pharmacokinetic profile—combining metabolic segregation with active central buffering—defines PG as a dual-mechanism delivery system that generates central neuroactive metabolites without disrupting endogenous neurosteroidogenesis, positioning it as a promising neurotherapeutic candidate minimizing physiological steroid homeostasis disruption.

The distinct mechanisms of progesterone and allopregnanolone neuroprotection have been demonstrated in mice variably expressing intracellular progesterone receptors (iPR). While progesterone requires iPR for neuroprotection, allopregnanolone exerts protection even in iPR-heterozygous mice, confirming an iPR-independent mechanism likely mediated through GABA_A_R modulation [45]. Importantly, neither progesterone nor allopregnanolone amplified post-stroke neurogenesis, indicating that their long-term benefits result from tissue preservation rather than neuronal replacement [45]. By delivering pregnanolone—a structural analog of allopregnanolone—directly via glutamate conjugation, PG bypasses the iPR requirement and achieves neuroprotection through direct GABA_A_R potentiation. This iPR-independent mechanism ensures consistent therapeutic efficacy regardless of individual variability in progesterone receptor expression, a critical advantage for translational application in heterogeneous human stroke populations.

## 4. Limitations of the Study

While this study provides a comprehensive metabolic profiling of pregnanolone glutamate (PG), several constraints regarding the experimental design should be considered when interpreting the results.

First, the experiments were conducted in immature rats (P12) to specifically model perinatal pathology. Given the significant developmental shifts in hepatic enzymatic activity and blood–brain barrier permeability, the specific metabolite ratios observed here may not directly extrapolate to adult physiology. However, this age group is the most relevant for the intended pediatric indications of PG.

Second, the study utilized a single time-point analysis (60 min post-administration). While this window effectively captures the rapid phase of distribution—evidenced by the robust accumulation of the parent compound in the hippocampus (69.3 pmol/g)—it provides a static snapshot of the pharmacological peak. Consequently, it does not fully resolve the elimination kinetics or the terminal half-life of the conjugated metabolites.

Third, the intraperitoneal route resulted in substantial hepatic exposure and metabolism. This is reflected in the high hepatic levels of conjugated metabolites (316 pmol/g vs. 184 pmol/g in serum; Table 3). While this route does not fully replicate the first-pass effect of oral administration, it appropriately emphasizes the liver’s role in generating the systemic metabolite profile.

Fourth, the study employed a tiered sampling strategy: while hippocampal analysis included the full cohort (n = 16 PG+, n = 27 PG–), comprehensive multi-tissue profiling (serum, hippocampus, liver, kidney) was performed on a representative subset (n = 6 PG+, n = 21 PG–). This approach was justified by the expectation of large effect sizes in peripheral metabolism based on preliminary data, allowing for a focused analytical strategy without compromising statistical power. Nevertheless, the smaller sample size for multi-tissue analysis may partly limit the detection of subtle tissue-specific differences in metabolite distribution.

Fifth, steroid concentrations were determined in whole-tissue homogenates. Although the strong intra-tissue correlations observed in OPLS analysis (e.g., R > 0.9 for pregnanolone and its metabolites) suggest tight metabolic coupling, this bulk analysis averages the steroid content across the tissue. It precludes the distinction between intracellular and extracellular pools or the identification of cell-specific uptake (e.g., neuronal vs. glial) within the hippocampus.

Sixth, while this study comprehensively characterizes the metabolic fate and tissue distribution of PG and its metabolites, it does not include direct neuroprotective endpoints (e.g., histological assessment of infarct volume, neuronal survival quantification, or functional behavioral testing). The inference of ‘neuroprotective potential’ is based on the known pharmacological activities of the detected metabolites—specifically, their established roles as GABA_A_R positive allosteric modulators and NMDAR inhibitors, as demonstrated in prior studies [4,16,17,36,37,38,39,46]. Consequently, the relationship between the specific metabolite concentrations observed in the ischemic hippocampus and the degree of actual neuroprotection remains correlational and inferential. Establishing a direct quantitative link between tissue steroid levels and neuroprotective outcomes would require combined steroidomic and histopathological analyses performed in the same animals, ideally across multiple time points and dose levels.

Despite these limitations, the robust statistical power for hippocampal analysis (n = 16–27), the comprehensive steroidomic coverage spanning major 5β- and 5α-metabolic pathways (>30 quantified steroids including free and conjugated forms), and the convergent evidence from univariate and multivariate analyses provide strong support for the proposed dual-fate mechanism and metabolic segregation of PG. The pharmacokinetic profile described here, combined with the neuroprotective efficacy demonstrated in prior studies using the same model [16,17], provides a compelling rationale for further translational development. These constraints define clear directions for future research, as outlined in Section 5.

An additional methodological consideration is that transcardial perfusion was not performed prior to tissue collection. Consequently, residual intravascular blood within the cerebral microvasculature may contribute to the measured hippocampal steroid concentrations. To estimate the magnitude of this possible confound, we applied published values for residual cerebral blood volume in unperfused rat brain. Under normothermic, normovolemic conditions, cerebral blood volume in the rat has been reported in the range of approximately 30–50 µL per gram of tissue, corresponding to 3–5% of total tissue volume [18]. Using the mean serum pregnanolone concentration measured in our study (404 pmol/mL) and this range of residual blood volumes (30–50 µL/g), the estimated blood-borne pregnanolone contamination would be 12.1–20.2 pmol per gram of hippocampal tissue. Relative to the total measured hippocampal pregnanolone concentration (69.3 pmol/g, Table 2), this vascular contribution accounts for 17–29% of the signal, while the remaining 71–83% represents genuine tissue accumulation.

It should also be noted that hippocampal pregnanolone concentrations are reported as 69.3 pmol/g in Table 2 (geometric mean, full cohort n = 16) and 107 pmol/g in Table 3 (median, multi-tissue subset n = 6). This difference reflects the use of different statistical measures (mean vs. median) and sample sizes rather than a biological inconsistency. Steroidomic data characteristically exhibit right-skewed distributions, particularly following pharmacological saturation of metabolic pathways. In such cases, the median may exceed the geometric mean when analyzing smaller subsets enriched for high-metabolizer phenotypes. Both values fell within the expected range for this experimental paradigm, and the ~170-fold increase relative to vehicle controls (0.41 pmol/g) was consistent across all analyses. For the blood contamination estimate above, we conservatively used the lower value (69.3 pmol/g from Table 2), which provides a more stringent test of the tissue accumulation hypothesis.

This substantial tissue-resident fraction, combined with the analyte-specific distribution gradients observed (e.g., hippocampal enrichment of 5β,20α-tetrahydroprogesterone relative to serum), confirms that residual intravascular blood is not the primary source of measured brain steroid levels. Moreover, the observed region-specific enrichment pattern—with hippocampal concentrations substantially exceeding those predicted from blood contamination alone—further supports active local accumulation or the retention of pregnanolone in hippocampal tissue rather than passive vascular carryover. Several observations further argue against blood contamination as a significant driver of our results. First, multiple analytes exhibited hippocampal concentrations substantially exceeding those predicted from residual blood volume alone (e.g., 5β,20α-tetrahydroprogesterone: 983 vs. 150 fmol/g in PG− rats; epipregnanolone: 132 vs. 14.5 fmol/g in PG− rats), a pattern incompatible with passive vascular carryover. Second, conjugated pregnanolone was markedly lower in the hippocampus than in serum (27.6 vs. 184 pmol/g in PG+ rats), demonstrating selective BBB restriction of polar conjugates that would not be observed if vascular contamination dominated the signal. Third, the OPLS intra-hippocampal analysis (Table 8) revealed a modular segregation between free and conjugated steroid pools—a tissue-specific metabolic architecture that cannot arise from simple dilution of the serum steroid profile. We also note that transcardial perfusion itself may introduce artifacts in steroidomic studies, including washout of loosely membrane-associated steroids, mechanical disruption of cellular compartments, and dilution of extracellular steroid pools. Our protocol of rapid dissection and immediate snap-freezing was chosen to minimize post-mortem metabolic changes, which we consider the priority for accurate steroidome profiling. Nevertheless, future studies employing controlled perfusion with steroid-free solutions, or complementary approaches such as in vivo microdialysis, would provide definitive resolution of the intravascular versus parenchymal steroid distribution.

## 5. Future Directions

The findings presented here establish a foundation for several critical lines of investigation that would substantially advance our understanding of pregnanolone glutamate pharmacology and its therapeutic optimization.

A comprehensive time-course study spanning early distribution (5–15 min), peak accumulation (30–60 min), and elimination phases (2–6 h) would resolve the full pharmacokinetic profile of both parent compound and conjugated metabolites. Such data are essential for determining optimal dosing intervals and predicting steady-state concentrations in repeated-dose regimens. Parallel investigation of age-dependent pharmacokinetics—comparing neonatal (P7), juvenile (P12), adolescent (P21), and adult rats—would clarify how developmental maturation of hepatic enzymes and blood–brain barrier transporters influence the metabolic fate of PG. This is particularly relevant given that current clinical trials target both neonatal hypoxic-ischemic encephalopathy and adult stroke populations.

The spatial resolution of metabolite distribution within the hippocampus represents another critical gap. Matrix-assisted laser desorption/ionization mass spectrometry imaging (MALDI-MSI) could map the regional heterogeneity of pregnanolone and its conjugates across CA1, CA3, and dentate gyrus subfields, potentially revealing subregion-specific vulnerabilities or therapeutic windows. Complementary approaches using immunohistochemistry with cell-type-specific markers would distinguish neuronal versus glial uptake, addressing whether the observed neuroprotection operates primarily through direct neuronal GABA_A_R modulation or involves astrocytic intermediates.

Route-of-administration studies comparing intravenous, intraperitoneal, and oral delivery would provide translational guidance for clinical formulation. While the present intraperitoneal model appropriately simulates hepatic first-pass metabolism, direct intravenous administration would isolate the contribution of peripheral metabolism from hepatic processing. Oral bioavailability studies in juvenile rats would be particularly valuable given the practical advantages of enteral administration in neonatal intensive care settings.

The mechanistic basis for the observed metabolic segregation—wherein brain-derived pregnanolone remains unconjugated while peripherally formed metabolites are extensively conjugated—warrants targeted investigation. In vitro studies using primary hippocampal cultures and hepatocyte co-cultures could test whether this reflects differential expression of sulfotransferases and UDP-glucuronosyltransferases, limited cofactor availability in neural tissue, or active efflux of conjugating enzymes. Transporter inhibition experiments (e.g., using probenecid to block organic anion transporters) would clarify whether conjugated metabolites are actively excluded from the brain or simply fail to cross the blood–brain barrier.

Additionally, structure–activity relationship studies exploring alternative conjugates (e.g., different amino acid esters or lipophilicity-modulated analogs) would enable rational optimization of the dual-fate delivery mechanism, potentially yielding derivatives with enhanced brain penetration or prolonged neuroprotective action.

The complex role of neurosteroid synthesis in perinatal brain injury warrants further investigation. Recent studies using the 5α-reductase inhibitor finasteride—which blocks the conversion of progesterone to allopregnanolone and pregnanolone—have revealed paradoxical outcomes in neonatal birth asphyxia models. Despite the expected reduction in GABAergic neurosteroid levels, finasteride administration unexpectedly decreased seizure severity and prevented cognitive impairment [47]. These findings suggest that endogenous neurosteroid dynamics following brain injury may differ substantially from exogenous supplementation, potentially involving compensatory upregulation of alternative neuroprotective pathways or differential temporal dynamics of neurosteroid synthesis. Measurement of brain tissue levels of pregnanolone, allopregnanolone, and their precursors at multiple time points following pregnanolone glutamate administration—combined with parallel finasteride co-treatment experiments—would clarify whether the therapeutic efficacy of PG depends primarily on direct neurosteroid delivery or also engages endogenous synthesis pathways. Such mechanistic insights would inform optimal combination strategies and identify patient populations most likely to benefit from neurosteroid-based interventions.

Finally, the functional consequences of the dual-fate metabolism require direct assessment. Electrophysiological recordings in hippocampal slices could compare the GABAergic potentiation induced by unconjugated pregnanolone versus its conjugated forms, testing whether sulfated and glucuronidated metabolites retain, lose, or acquire distinct receptor activities. Behavioral studies examining anxiolytic, anticonvulsant, and neuroprotective endpoints in models with genetically or pharmacologically altered conjugation capacity would establish whether peripheral metabolism represents a detoxification pathway or generates bioactive species with independent therapeutic value.

Critically, future studies should directly correlate tissue steroid concentrations with neuroprotective outcomes measured in the same animals. This could be achieved by combining the steroidomic profiling approach used here with quantitative histopathological assessment (e.g., Fluoro-Jade B staining for degenerating neurons, NeuN immunohistochemistry for neuronal survival, TUNEL assay for apoptosis) and functional behavioral testing (e.g., Morris water maze for spatial memory, rotarod for motor coordination) at matched time points. Dose–response studies incorporating multiple PG doses (e.g., 0.25, 0.5, 1.0, and 2.0 mg/kg) with parallel steroidomic and histological endpoints would enable the construction of concentration–effect relationships, directly linking specific hippocampal metabolite thresholds to neuroprotective efficacy. Such data would transform the pharmacokinetic framework established here into a quantitative pharmacokinetic-pharmacodynamic (PK-PD) model with direct translational applicability.

Collectively, these investigations would transform the current snapshot of PG metabolism into a dynamic, mechanistically grounded framework capable of guiding rational drug development and personalized dosing strategies for neurosteroid-based therapeutics.

## 6. Materials and Methods

### 6.1. Animals

Experiments were performed on male and female Wistar albino rats (bred by the National Institute of Mental Health, Czech Republic, Approval No. MZE-14903/2025-13143) at P12. To optimize resource allocation, hippocampal tissue (the primary target organ) was analyzed in a larger cohort (n = 16 PG+, n = 27 PG−) to adequately capture biological variability. Complete multi-tissue sets including serum, hippocampus, liver, and kidney were collected from a representative subgroup (n = 6 PG+, n = 21 PG−), where effect sizes for peripheral metabolism were expected to be large based on preliminary data. The day of birth was defined as Day 0. Rats were housed in a controlled environment (temperature 22 ± 1 °C, humidity 50–60%, lights on from 6 a.m. to 6 p.m.) with free access to food and water. During the experiments, the temperature in plexiglass cages was maintained at the temperature of the nest (34.5 ± 0.5 °C) using an electric heating pad connected to a digital thermometer to compensate for the immature thermoregulatory function at this age [48]. All procedures involving animals and their care were conducted according to the ARRIVE guidelines https://www.nc3rs.org.uk/arrive-guidelines (access date 28 November 2021) in compliance with national (Act No. 246/1992 Coll.) and international laws and policies (EU Directive 2010/63/EU for animal experiments and the National Institutes of Health Guide for the Care and Use of Laboratory Animals, NIH Publication No. 8023, revised 1978). The experimental protocol was approved by the Ethical Committee of the National Institute of Mental Health and the Ministry of Health Animal Care and Use Committee (project proposal approval No. MZDR 2240/2025/OVZ).

### 6.2. Pregnanolone Glutamate

The compound pregnanolone glutamate (20-oxo-5β-pregnan-3α-yl L-glutamyl 1-ester, PG) was synthesized according to the literature [46,49]. PG was dissolved in a solution of 3 g of (2-hydroxypropyl)-β-cyclodextrin (CDX, Sigma-Aldrich, St. Louis, MO, USA) and 157 mg of citric acid (Sigma-Aldrich, St. Louis, MO, USA) in 30 mL of distilled water. The pH was adjusted to 7.36 with NaOH (Sigma-Aldrich, St. Louis, MO, USA).

### 6.3. Induction of Perinatal Focal Cerebral Ischemia (pFCI) by Endothelin-1 (ET-1)

Twelve-day-old male and female Wistar rats were used in all experiments. Perinatal focal cerebral ischemia (pFCI) was induced by the intrahippocampal infusion of endothelin-1 (ET-1) at a concentration of 40 pmol in a total volume of 1 μL into the right dorsal hippocampus according to the procedure described by [50]. Control animals received intrahippocampal infusion of phosphate-buffered saline (PBS) (pH 7.4) in a total volume of 1 μL.

Surgery was performed under isoflurane anesthesia (1.5–2.0%) as described previously [50,51,52]. Briefly, an internal cannula for drug infusion was stereotaxically inserted into the right dorsal hippocampus (stereotaxic coordinates anteroposterior (AP) = 3.5 mm, mediolateral (ML) = 3.0 mm, dorsoventral (DV) = 3.5 mm). Coordinates were calculated for each animal according to their bregma-lambda distance to adjust for immature brain size [53]. Drug or control solution was infused at a constant speed of 0.25 μL/min using a pulse-free pump (kdS #789200W, WPI (World Precision Instruments), Sarasota, FL, USA) for 4 min. After the infusion, the skin was closed with non-toxic glue (Collodium, Penta, Prague, Czech Republic), and the rat was removed from the stereotaxic device and immediately placed into a transparent Plexiglas chamber (dimensions: 50 × 25 × 25 cm, L × W × H) positioned on a heating pad maintained at 34.5 ± 0.5 °C to replicate nest temperature and ensure thermoregulation during recovery. The total duration of the surgical procedure corresponded to the time spent under anesthesia and did not exceed 14 ± 1 min.

Animals were randomly assigned to two experimental groups: PG+ rats (n = 16) and PG− rats (n = 27). Pregnanolone glutamate (PG) was dissolved in β-cyclodextrin (CDX) as previously reported by Kleteckova et al. (2014) [16]. Five minutes after the end of intrahippocampal infusion, rats received either PG (1 mg dissolved in 4 mL vehicle) or the vehicle alone (4 mL CDX) via intraperitoneal injection. Given the body weight range of 25–30 g at P12, the injection volume was calculated as 40 μL per 10 g of body weight, resulting in individual doses of 100–120 μL per animal. Throughout the experiments, animals were maintained under nest-like thermal conditions (34.5 ± 0.5 °C).

### 6.4. Tissue Samples for Metabolomic Analysis

The experimental groups included PG^−^ rats with intrahippocampal infusion of PBS and intraperitoneal administration of CDX (n = 27), and PG+ rats with intrahippocampal infusion of endothelin-1 and intraperitoneal administration of PG (n = 16). Animals were sacrificed 60 min after drug administration. Blood was collected via cardiac puncture, and hippocampi were rapidly dissected, weighed, and frozen at −80 °C in sterile plastic microvials until analysis. Transcardial perfusion was not performed prior to tissue collection. This decision was made to avoid potential perfusion-induced artifacts in steroid quantification, including washout of membrane-associated and loosely bound steroids, and to preserve the native steroidomic profile of the tissue. The potential contribution of residual intravascular blood to measured tissue concentrations is addressed quantitatively in Section 4 (Limitations). In a representative subset (n = 6 PG+, n = 21 PG−), liver and kidney tissues were additionally collected to enable comprehensive multi-tissue steroidome profiling (Table 5, Table 6 and Table 7). This tiered sampling strategy was justified by the expected large effect sizes in peripheral metabolism, allowing focused analytical resources while maintaining adequate statistical power.

### 6.5. Steroid Analysis

Steroids and their polar conjugates were measured using our previously described, validated GC-MS/MS method [54].

Steroids were included in the primary hippocampal analysis (Table 2, Table 3 and Table 4) if they met the following criteria: (1) CV < 60% within treatment groups, (2) values consistently above the limit of quantification (LOQ), and (3) statistical power >0.7 for detecting between-group differences at α = 0.05. Free epipregnanolone in the hippocampal tissue exhibited high inter-individual variability (CV ≥ 82%) and was therefore reported only in the tissue distribution analysis (Table 3, multi-tissue subset n = 6 PG+, n = 21 PG−) but excluded from the main hippocampal dataset (Table 2, full cohort n = 16 PG+, n = 27 PG−) and correlation matrices (Tables S2 and S5).

### 6.6. Statistical Analysis

In the first step, the power transformation parameters were found for each metric variable so that its distribution was as close as possible to the Gaussian distribution. The steroidomic data were evaluated using an ANOVA model as well as multivariate regression with reduced dimensionality known as orthogonal projections to latent structure (OPLS) model. Due to the dependence on age for many of the steroidomic data, besides the Subject factor (individual animals), the ANOVA model included the between-subject factors PG (PG+ rats vs. PG− rats), within-subject factor biological material (BM), comprising serum (SER), right hippocampus (RH), liver (LI), and kidney (KI) and PG × BM interaction. Statgraphics Centurion version XVIII statistical software from Statgraphics Technologies, Inc. (The Plains, VA, USA) was used for power transformations of the original data and for evaluation using the ANOVA model, while SIMCA-P version 12.0 statistical software from Umetrics AB (Umeå, Sweden) was used for OPLS analysis.

Separate OPLS models were constructed for PG+ and PG− rats to investigate relationships between individual 5β-steroids in the right hippocampus and remaining hippocampal and all serum 5β-steroids. The OPLS model, which is a multivariate regression with dimensionality reduction, permits the evaluation of relationships between explanatory variables and several explanatory variables that may be highly correlated, which is also the case for steroids in metabolic pathways [55].

The variability of the explanatory and response variables is separated into two independent components in the OPLS. The former contains the variability in explanatory variables that were shared with the probability of pathology (predictive component), while the orthogonal components express the variability shared among highly correlated explanatory variables (orthogonal components). OPLS identifies significant explanatory variables and their best linear combination to estimate the probability of the presence of pathology. After standardizing the variables, the OPLS model can be expressed as follows:

(1) $$X=T_{p}P_{p}^{T}+T_{0}P_{0}^{T}+E$$

(2) $$Y=T_{p}P_{p}^{T}+F$$

where X is the matrix with explanatory variables and subjects, Y is the vector of dependent variable and subjects; $T_{p}$ is the vector of component scores from the single predictive component and subjects extracted from Y; $T_{0}$ is the vector of component scores from the single orthogonal component and subjects extracted from X; $P_{p}$ is the vector of component loadings for the predictive component extracted from Y; $P_{0}$ is the vector of component loadings for the orthogonal component extracted from X and independent variables; and E and F are the error terms.

Significant explanatory variables were selected using the Variable Importance in Projection statistics (VIP). The statistical software SIMCA-P v.12.0, which was used for OPLS analysis, enabled finding the number of relevant components, the detection of multivariate non-homogeneities, and testing the multivariate normal distribution and homoscedasticity (constant variance).

The algorithm for obtaining the predictions was as follows:

- Transformation of the original data to obtain values with a symmetric distribution and constant variance.
- Checking data homogeneity in explanatory variables using Hotelling’s statistics and the eventual elimination of outliers.
- Testing the relevance of explanatory variables using Variable Importance in Projection (VIP) statistics and the elimination of irrelevant explanatory variables.
- Calculating component loadings for individual variables to evaluate their correlations with the predictive component.
- Calculating regression coefficients for the multiple regression model to evaluate the mutual independence of explanatory variables after comparison with the corresponding component loadings from the OPLS model.
- Calculating predicted values of the dependent variable (concentration of the specific hippocampal 5β-steroid).
- Evaluating model performance by calculating the correlation coefficient between observed and predicted values (multiple correlation coefficient) and the proportion of explained variance (R^2^).

To address the multiplicity arising from simultaneous testing of numerous steroid analytes, two complementary correction strategies were employed. First, for all post hoc pairwise comparisons of biological materials (Table 5 and Table 6), Bonferroni correction was applied separately for the PG+ and PG− groups to control the family-wise error rate. With 4 tissues (serum, hippocampus, liver, kidney) yielding 6 pairwise comparisons per group, the corrected significance threshold was set at α = 0.0083 (0.05/6). Second, for the primary hippocampal analyses (Table 2 and Table 4), where multiple analytes were tested for the main effect of PG treatment, the Benjamini–Hochberg False Discovery Rate (FDR) procedure was applied across all analytes within each table to control the expected proportion of false discoveries at q < 0.05. FDR-adjusted q-values are reported alongside nominal *p*-values in the revised tables. Analytes reaching nominal significance (*p* < 0.05) but failing FDR correction (q ≥ 0.05) are flagged as exploratory findings. For the OPLS multivariate models (Table 7 and Table 8), multiplicity is inherently controlled through dimensionality reduction onto latent components, the proportion of explained variance for the cross-validated model (Q^2^), and Variable Importance in Projection (VIP) thresholds, which collectively guard against overfitting and spurious associations.

## 7. Conclusions

This study provides the first comprehensive pharmacokinetic characterization of pregnanolone glutamate (PG) in a perinatal focal cerebral ischemia model, revealing a unique dual-fate mechanism that distinguishes PG from conventional neurosteroid therapeutics.

Our findings establish that PG functions as a sophisticated “molecular shuttle” rather than a simple prodrug. Following systemic administration, the tissue distribution pattern at 60 min is consistent with extensive peripheral hydrolysis of PG by γ-glutamyl hydrolase (GGH) [17], generating lipophilic pregnanolone that crosses the blood–brain barrier and accumulates in the ischemic hippocampus. The temporal dynamics of this process require confirmation through a dedicated multi-time-point pharmacokinetic study. Simultaneously, a fraction of intact PG enters the CNS via carrier-mediated transport [14,17,21,22,23,24], creating a sustained reservoir of the polar conjugate. This parallel influx mechanism ensures both immediate and prolonged delivery of neuroactive steroids to the injured brain.

Quantitative steroidome profiling demonstrates that neuroprotection is mediated predominantly through GABA_A_R potentiation by pregnanolone and its metabolites (particularly 17-hydroxypregnanolone) [16,17,36], with NMDA receptor antagonism by intact PG playing a secondary role during acute excitotoxicity [3,4]. The steep serum-to-hippocampus gradient for free pregnanolone (404→107 pmol/g) versus limited penetration of the conjugate (42.3 pmol/g) confirms that GABAergic modulation—not direct NMDA blockade—constitutes the primary therapeutic mechanism.

Critically, PG administration induces “metabolic segregation” within the CNS: the exogenous 5β-pathway saturates (~170-fold increase in pregnanolone), while the endogenous 5α-pathway (allopregnanolone) remains undisturbed (*p* = 0.411). This selective saturation preserves the brain’s intrinsic neuroprotective machinery [37,38,39], distinguishing PG from direct neurosteroid administration that may disrupt physiological homeostasis. Furthermore, preferential hippocampal accumulation of 3β-isomers and oxidized metabolites suggests active intracerebral buffering via HSD17B enzymes [40], providing an autonomous safety mechanism against excessive GABAergic inhibition.

The brain functions as a “metabolic sink” for peripherally generated metabolites [17], passively accumulating compounds such as 17-hydroxypregnanolone and etiocholanolone—products of hepatic CYP17A1 activity absent in neural tissue. This multi-tissue metabolic cooperation enables PG to deliver a polypharmacological cocktail of metabolites with established GABAergic modulatory activity [17,36,37,38,39] without requiring local synthesis, thereby circumventing the metabolic limitations of the immature or injured brain. While the present study characterizes this pharmacokinetic delivery mechanism, the neuroprotective efficacy of PG in the same pFCI model has been independently established [16,17].

In summary, pregnanolone glutamate represents a paradigm shift in neurosteroid therapeutics. By combining peripheral prodrug activation with direct CNS delivery, metabolic pathway selectivity with endogenous homeostasis preservation, and polypharmacological metabolite generation with intrinsic safety buffering, PG offers a superior pharmacokinetic profile for treating perinatal hypoxic-ischemic brain injury. Together with the previously demonstrated neuroprotective efficacy in the same experimental model [16,17], these pharmacokinetic findings position PG as a promising clinical candidate that harnesses the body’s own metabolic machinery to achieve targeted, sustained, and self-regulated delivery of neuroprotective steroids to the injured brain.

Future translational studies should focus on age-dependent pharmacokinetics, route-of-administration optimization, and the therapeutic window for PG intervention in neonatal encephalopathy, with the ultimate goal of establishing this dual-mechanism neurosteroid as a first-line treatment for perinatal brain injury.

## Figures and Tables

**Table 1:** Physicochemical properties of pregnanolone forms.

Compound	Lipophilicity	Polarity	BBB Penetration	Main Metabolites	Key References
Pregnanolone	High	Low	Easy, rapid	Hydroxylated androstanes (e.g., etiocholanolone)	[17]
Pregnanolone Glutamate	Moderate	High	Limited, possibly intact transport	Pregnanolone + glutamate (after hydrolysis)	[17]
Pregnanolone Sulfate	Low	High	Restricted, transporter-dependent	Desulfated pregnanolone (via steroid sulfatase (STS) activity)	[14,15]

**Table 7:** OPLS/MR relationships between serum 5β-steroids (explanatory variables) and hippocampal 5β-steroids (response variables).

**Serum Steroids**	Steroids in Right Hippocampus (Response Variables)											
(explanatory variables)	Pregnanolone		Pregnanolone, C		Epipregnanolone		Epipregnanolone, C		17-Hydroxy-		17-Hydroxy-	
									pregnanolone		pregnanolone, C	
	PG+	PG−	PG+	PG−	PG+	PG−	PG+	PG−	PG+	ns	PG+	PG−
	0.9 **	0.594 *	0.772 **	ns	ns	ns	0.74 *	0.656 **	0.772 **	ns	ns	ns
Pregnanolone	0.88 2**/*	0.878 **/**	0.61 */*	ns	ns	ns	ns	ns	ns	ns	ns	ns
Pregnanolone, C	0.953 **/*	ns	−0.884 **/**	ns	ns	ns	ns	−0.696 */*	ns	ns	ns	ns
Epipregnanolone	0.985 **/**	ns	0.528 **/**	ns	ns	ns	ns	ns	ns	ns	ns	ns
Epipregnanolone, C	0.673/ *	0.7 **/*	0.819 */*	ns	ns	ns	ns	ns	ns	ns	ns	ns
17-Hydroxypregnanolone	ns	0.728 **/**	ns	ns	ns	ns	−0.985 **/**	ns	0.986 **/*	ns	ns	ns
17-Hydroxypregnanolone, C	ns	ns	ns	ns	ns	ns	−0.949 **/**	−0.718 */*	0.938 **/*	ns	ns	ns
5β,20α-Tetrahydroprogesterone	0.889 **/**	ns	0.744 */*	ns	ns	ns	ns	ns	ns	ns	ns	ns
5β,20α-Tetrahydroprogesterone, C	ns	ns	ns	ns	ns	ns	ns	ns	−0.78 */	ns	ns	ns
5β-Pregnane-3α,20α-diol	ns	0.767 **/*	ns	ns	ns	ns	−0.988 **/**	ns	0.982 **/*	ns	ns	ns
5β-Pregnane-3α,20α-diol, C	0.846 */*	ns	0.79 **/*	ns	ns	ns	ns	ns	ns	ns	ns	ns
5β-Pregnane-3β,20α-diol	ns	ns	0.713 */**	ns	ns	ns	ns	0.652 */*	ns	ns	ns	ns
5β-Pregnane-3β,20α-diol, C	ns	ns	ns	ns	ns	ns	ns	ns	ns	ns	ns	ns
5β-Pregnane-3α,17,20α-triol	ns	−0.358/ *	ns	ns	ns	ns	−0.97 **/**	0.496 */*	0.969 **/**	ns	ns	ns
Etiocholanolone	0.809 */*	ns	ns	ns	ns	ns	ns	ns	ns	ns	ns	ns
Etiocholanolone, C	ns	ns	ns	ns	ns	ns	−0.99 **/**	ns	0.978 **/**	ns	ns	ns
Epietiocholanolone	0.721 */*	ns	ns	ns	ns	ns	ns	ns	ns	ns	ns	ns
Epietiocholanolone, C	ns	ns	ns	ns	ns	ns	ns	ns	ns	ns	ns	ns
5β-Androstane-3α,17β-diol, C	ns	ns	ns	ns	ns	ns	ns	ns	ns	ns	ns	ns
(explanatory variables)	5β,20α-Tetrahydro-		5β,20α-Tetrahydro-		5β-Pregnane-		5β-Pregnane-		5β-Pregnane-		5β-Pregnane-	
	progesterone		progesterone, C		3α,20α-diol		3α,20α-diol, C		3β,20α-diol		3β,20α-diol, C	
	PG+	PG−	PG+	PG−	PG+	PG−	PG+	PG−	PG+	PG−	PG+	PG−
	0.65 */	0.651 **/	0.786 **/	ns	0.819 */	0.756 */	ns	ns	ns	0.755 **/	ns	ns
Pregnanolone	−0.785 */	0.76 **/*	ns	ns	ns	ns	ns	ns	ns	ns	ns	ns
Pregnanolone, C	−0.961 **/*	ns	−0.975 **/**	ns	ns	ns	ns	ns	ns	ns	ns	ns
Epipregnanolone	−0.978 **/	ns	−0.909 **/*	ns	ns	ns	ns	ns	ns	ns	ns	ns
Epipregnanolone, C	−0.799 */	ns	ns	ns	ns	ns	ns	ns	ns	ns	ns	ns
17-Hydroxypregnanolone	ns	ns	ns	ns	ns	ns	ns	ns	ns	ns	ns	ns
17-Hydroxypregnanolone, C	ns	ns	ns	ns	ns	0.861 **/**	ns	ns	ns	ns	ns	ns
5β,20α-Tetrahydroprogesterone	ns	ns	ns	ns	ns	ns	ns	ns	ns	ns	ns	ns
5β,20α-Tetrahydroprogesterone, C	ns	ns	ns	ns	ns	ns	ns	ns	ns	0.811 **/*	ns	ns
5β-Pregnane-3α,20α-diol	ns	ns	ns	ns	ns	0.569 */**	ns	ns	ns	ns	ns	ns
5β-Pregnane-3α,20α-diol, C	−0.926 **/	−0.926 **/	−0.925 **/**	ns	ns	−0.644 **/**	ns	ns	ns	ns	ns	ns
5β-Pregnane-3β,20α-diol	ns	ns	−0.841 **/*	ns	0.952 **/**	ns	ns	ns	ns	0.538/	ns	ns
5β-Pregnane-3β,20α-diol, C	ns	ns	ns	ns	ns	ns	ns	ns	ns	ns	ns	ns
5β-Pregnane-3α,17,20α-triol	ns	ns	ns	ns	ns	ns	ns	ns	ns	ns	ns	ns
Etiocholanolone	ns	−0.669/	ns	ns	ns	ns	ns	ns	ns	0.399 */	ns	ns
Etiocholanolone, C	ns	ns	ns	ns	ns	ns	ns	ns	ns	ns	ns	ns
Epietiocholanolone	ns	−0.652 */	−0.861 **/*	ns	0.888 **/	ns	ns	ns	ns	ns	ns	ns
Epietiocholanolone, C	ns	ns	ns	ns	ns	ns	ns	ns	ns	ns	ns	ns
5β-Androstane-3α,17β-diol, C	ns	ns	ns	ns	ns	ns	ns	ns	ns	ns	ns	ns
(explanatory variables)	5β-Pregnane-		Etiocholanolone		Etiocholanolone, C		Epietiocholanolone		Epietiocholanolone, C		5β-Androstane-	
	3α,17,20α-triol										3α,17β-diol, C	
	PG+	PG−	PG+	PG−	PG+	PG−	PG+	PG−	PG+	PG−	PG+	PG−
	0.998 **/	ns	ns	0.505 */	0.922 **/	ns	ns	0.567 */	0.953 **/	ns	0.998 **/	0.534 **/
Pregnanolone	ns	ns	ns	0.879 **/	ns	ns	ns	ns	ns	ns	ns	0.883 **/*
Pregnanolone, C	ns	ns	ns	ns	ns	ns	ns	ns	ns	ns	−0.716 **/**	0.829 **/**
Epipregnanolone	ns	ns	ns	ns	ns	ns	ns	ns	ns	ns	−0.635 **/*	ns
Epipregnanolone, C	ns	ns	ns	ns	ns	ns	ns	ns	ns	ns	ns	0.442/
17-Hydroxypregnanolone	0.792 **/**	ns	ns	ns	−0.978 **/**	ns	ns	ns	0.92 **/**	ns	ns	ns
17-Hydroxypregnanolone, C	ns	ns	ns	ns	−0.943 **/**	ns	ns	−0.678 */*	−0.903 **/**	ns	ns	ns
5β,20α-Tetrahydroprogesterone	ns	ns	ns	ns	ns	ns	ns	0.927 **/**	ns	ns	ns	ns
5β,20α-Tetrahydroprogesterone, C	ns	ns	ns	ns	ns	ns	ns	ns	ns	ns	ns	ns
5β-Pregnane-3α,20α-diol	0.611 **/**	ns	ns	0.953 **/**	−0.98 **/**	ns	ns	ns	−0.707 **/**	ns	ns	ns
5β-Pregnane-3α,20α-diol, C	ns	ns	ns	ns	ns	ns	ns	ns	ns	ns	ns	ns
5β-Pregnane-3β,20α-diol	ns	ns	ns	ns	−0.556/	ns	ns	ns	−0.688 */	ns	−0.891 **/**	0.264/
5β-Pregnane-3β,20α-diol, C	ns	ns	ns	ns	ns	ns	ns	ns	ns	ns	ns	ns
5β-Pregnane-3α,17,20α-triol	0.83 **/**	ns	ns	ns	−0.962 **/**	ns	ns	ns	−0.734 **/**	ns	ns	ns
Etiocholanolone	ns	ns	ns	ns	ns	ns	ns	ns	ns	ns	ns	ns
Etiocholanolone, C	0.772 **/**	ns	ns	ns	−0.988 **/**	ns	ns	ns	−0.864 **/**	ns	ns	ns
Epietiocholanolone	ns	ns	ns	ns	ns	ns	ns	ns	ns	ns	−0.897 **/**	−0.676 **/**
Epietiocholanolone, C	ns	ns	ns	ns	ns	ns	ns	ns	ns	ns	ns	ns
5β-Androstane-3α,17β-diol, C	ns	ns	ns	ns	ns	ns	ns	ns	ns	ns	ns	ns

Note: Serum-to-hippocampus analysis performed on a representative subset with complete collection of all biological materials (n = 6 PG+, n = 21 PG−), optimized for peripheral-to-central coupling analysis. Values represent correlation coefficients with a shared predictive component. Statistical significance is reported in the format OPLS/MR, where symbols before the slash refer to OPLS and symbols after the slash refer to multiple regression (MR). * *p* < 0.05, ** *p* < 0.01; ns, not significant.

**Table 8:** OPLS/MR relationships for individual 5β-steroids in right hippocampus vs. other hippocampal 5β-steroids.

Steroids, Explanatory Variables	Steroids, Response Variables											
	Pregnanolone		Pregnanolone, C		Epipregnanolone, C		17-Hydroxy- pregnanolone		17-Hydroxy- pregnanolone, C		5β,20α-Tetrahydroprogesterone	
	PG+	PG−	PG+	PG−	PG+	PG−	PG+	PG−	PG+	PG−	PG+	PG−
	0.949 **/	0.683 **/	0.912 **/	0.715 **/	0.923 **/	0.764 **/	0.976 **/	0.609 **/	0.969 **/	0.613 **/	0.894 **/	0.828 **/
Pregnanolone	-----	-----	−0.426 **/	ns	ns	ns	0.721 */*	0.766 **/*	−0.805 **/**	ns	0.811 **/**	0.387 */**
Pregnanolone, C	−0.436 **/	ns	-----	-----	ns	0.825 **/**	−0.821 **/**	ns	0.784 **/**	0.719 **/**	−0.854 **/**	−0.698 **/**
Epipregnanolone, C	ns	ns	0.518 */	0.644 **/**	-----	-----	−0.685/ *	ns	ns	ns	ns	−0.54 **/*
17-Hydroxypregnanolone	0.735 **/	0.777 **/**	−0.605 **/	ns	−0.508 */	ns	-----	-----	−0.911 **/**	ns	0.794 **/**	0.625 **/*
17-Hydroxypregnanolone, C	−0.604 **/	ns	0.826 **/**	0.516 **/**	0.633 */	ns	−0.922 **/**	ns	-----	-----	−0.859 **/**	−0.551 **/**
5β,20α-Tetrahydroprogesterone	0.845 **/*	0.864 **/**	−0.694 **/	−0.754 **/**	−0.323 **/*	ns	0.838 **/**	0.852 **/*	−0.886 **/**	−0.828 **/**	-----	-----
5β,20α-Tetrahydroprogesterone, C	0.607 */	ns	ns	ns	ns	ns	ns	ns	−0.588 */*	ns	0.634 */	ns
5β-Pregnane-3α,20α-diol	0.946 **/**	0.839 **/*	−0.332 */*	−0.692 */*	ns	ns	0.714 **/**	0.872 **/*	−0.78 **/**	−0.645 **/**	0.745 **/**	0.758 **/**
5β-Pregnane-3α,20α-diol, C	−0.512 */	ns	0.909 **/*	ns	0.686 **/	ns	−0.78 **/**	ns	0.796 **/**	0.628 **/**	−0.865 **/**	−0.464 */*
5β-Pregnane-3β,20α-diol	0.671 */*	ns	−0.524 */	ns	ns	ns	0.734 **/**	ns	ns	ns	0.698 **/**	ns
5β-Pregnane-3β,20α-diol, C	ns	ns	ns	ns	ns	0 **	ns	ns	ns	ns	ns	ns
5β-Pregnane-3α,17,20α-triol	ns	0.769 **/*	ns	−0.616 **/	ns	−0.608 */*	ns	ns	ns	ns	ns	0.671 */**
Etiocholanolone	ns	ns	ns	ns	−0.504/	ns	0.551 */*	ns	ns	ns	0.464 */*	ns
Etiocholanolone, C	−0.63 **/	ns	0.683 **/	0.741 **/**	0.799 **/**	ns	−0.938 **/**	ns	0.908 **/**	0.73 **/*	−0.763 **/**	−0.652 **/**
Epietiocholanolone	ns	ns	−0.748 **/**	ns	−0.359 **/	ns	0.564 **/**	ns	−0.45 */*	−0.433 */**	0.522 **/**	0.436 */*
Epietiocholanolone, C	ns	ns	ns	0.713 **/*	0.878 **/**	0.76 **/**	−0.659 */*	ns	0.529 */*	ns	ns	−0.498 **/*
5β-Androstane-3α,17β-diol, C	ns	ns	ns	0.716 **/*	0.831 **/	ns	−0.787 **/**	ns	0.676 **/*	0.684 **/*	ns	−0.583 **/**
Steroids, explanatory variables	Steroids, response variables											
	5β,20α-Tetrahydro- progesterone, C		5β-Pregnane- 3α,20α-diol		5β-Pregnane- 3α,20α-diol, C		5β-Pregnane- 3β,20α-diol		5β-Pregnane- 3β,20α-diol, C		5β-Pregnane- 3α,17,20α-triol	
	PG+	PG−	PG+	PG−	PG+	PG−	PG+	PG−	PG+	PG−	PG+	PG−
	0.895 **/	0.591 */	0.551 **/	0.729 **/	0.936 **/	0.711 **/	0.755 **/	ns	0.519 **/	0.786 **/	0.753 **/	0.731 **/
Pregnanolone	0.845 **/*	ns	0.721 **/**	0.446 **/**	−0.528 **/	ns	0.768 **/*	ns	ns	ns	ns	0.362 */*
Pregnanolone, C	−0.788 **/**	ns	−0.821 **/**	−0.685 **/*	0.938 **/**	0.816 **/**	−0.802 **/*	ns	ns	ns	ns	−0.714 **/
Epipregnanolone, C	ns	ns	ns	−0.549 **/*	ns	0.579 */**	ns	ns	ns	ns	−0.859 */*	−0.6 **/*
17-Hydroxypregnanolone	ns	ns	−0.685 **/*	0.657 **/*	−0.524 **/	ns	0.836 **/*	ns	−0.742 **/*	ns	0.873 **/*	0.61 */
17-Hydroxypregnanolone, C	−0.856 **/**	ns	−0.922 **/	−0.49 **/**	0.882 **/**	0.662 **/**	ns	ns	0.838 **/*	ns	−0.69 */	ns
5β,20α-Tetrahydroprogesterone	0.884 **/*	ns	0.838 **/*	0.871 **/**	−0.747 **/	−0.75 **/*	0.879 **/**	ns	ns	ns	ns	0.81 **/*
5β,20α-Tetrahydroprogesterone, C	-----	-----	ns	ns	−0.715 **/*	ns	0.567 */*	ns	ns	0.686 **/**	ns	ns
5β-Pregnane-3α,20α-diol	0.752 **/*	ns	-----	-----	−0.404 */	−0.716 **/*	0.689 **/*	ns	ns	ns	0.562 */*	0.775 **/**
5β-Pregnane-3α,20α-diol, C	−0.848 **/**	−0.443/	0.714 **/	−0.441 */*	-----	-----	−0.818 **/**	ns	ns	ns	−0.545/	ns
5β-Pregnane-3β,20α-diol	0.649 */*	0.442/ *	−0.78 **/*	ns	−0.494 */	ns	-----	-----	ns	ns	ns	ns
5β-Pregnane-3β,20α-diol, C	ns	0.738 **/**	ns	ns	ns	0.411 */**	ns	ns	-----	-----	ns	ns
5β-Pregnane-3α,17,20α-triol	ns	ns	ns	0.707 **/**	ns	ns	ns	ns	ns	ns	-----	-----
Etiocholanolone	ns	ns	ns	ns	−0.372/	ns	0.52 */**	ns	−0.546 */*	0.365 */	0.785/	ns
Etiocholanolone, C	ns	ns	0.734 */	−0.614 **/	0.63 **/	0.733 **/**	ns	ns	0.929 **/	0.383 **/*	ns	−0.711 **/**
Epietiocholanolone	ns	ns	ns	ns	−0.564 **/*	ns	0.609 **/**	ns	ns	0.572 */**	0.67/	ns
Epietiocholanolone, C	ns	ns	ns	−0.494 **/*	ns	ns	ns	ns	ns	ns	−0.728 **/**	−0.614 */**
5β-Androstane-3α,17β-diol, C	ns	ns	ns	ns	ns	0.763 **/**	ns	ns	0.867 **/*	0.5 */	−0.826 **/*	−0.607 **/*
Steroids, explanatory variables		Steroids, response variables										
		Etiocholanolone		Etiocholanolone, C		Epietiocholanolone		Epietiocholanolone, C		5β-Androstane-3α,17β- diol, C		
		PG+	PG−	PG+	PG−	PG+	PG−	PG+	PG−	PG+	PG−	
		0.924 **/	0.789 **/	0.964 **/	0.77 **/	0.974 **/	0.806 **/	0.916 **/	0.7 **/	0.899 **/	0.868 **/	
Pregnanolone		ns	0.399/	−0.565 **/	ns	ns	ns	ns	−0.034/	ns	ns	
Pregnanolone, C		−0.489 */	ns	0.647 **/	0.746 **/**	−0.555 **/	ns	ns	0.792 **/*	ns	0.67 **/	
Epipregnanolone, C		ns	ns	0.825 */**	ns	ns	ns	0.892 **/**	0.651 **/**	0.896 **/*	ns	
17-Hydroxypregnanolone		0.592 */	ns	−0.712 **/	ns	0.504 **/	ns	ns	ns	−0.601 */	ns	
17-Hydroxypregnanolone, C		ns	ns	0.803 **/	0.573 **/*	−0.404 */**	−0.522 */*	ns	ns	0.743 */	ns	
5β,20α-Tetrahydroprogesterone		ns	ns	−0.648 **/*	−0.66 **/*	0.503 **/*	0.721 **/**	ns	−0.654 **/*	−0.468 **/	−0.415 */	
5β,20α-Tetrahydroprogesterone, C		ns	ns	ns	ns	ns	ns	ns	ns	ns	ns	
5β-Pregnane-3α,20α-diol		ns	0.152/	−0.662 **/*	ns	ns	ns	ns	−0.605 */*	−0.593 */	ns	
5β-Pregnane-3α,20α-diol, C		ns	ns	0.557 */	ns	−0.506 */	ns	ns	ns	ns	ns	
5β-Pregnane-3β,20α-diol		0.601 **/	ns	ns	ns	0.681 **/	ns	ns	ns	ns	ns	
5β-Pregnane-3β,20α-diol, C		0.77 **/*	ns	ns	ns	ns	0.316/ *	ns	ns	0.628 **/**	0.607 **/**	
5β-Pregnane-3α,17,20α-triol		0.886 */**	ns	ns	ns	ns	ns	ns	−0.58 **/*	ns	ns	
Etiocholanolone		-----	-----	ns	ns	0.874 */**	0.765 **/**	ns	ns	ns	ns	
Etiocholanolone, C		ns	0.57 */**	-----	-----	ns	ns	0.935 **/*	0.815 **/**	0.941 **/**	0.836 **/**	
Epietiocholanolone		ns	0.724 **/**	ns	ns	-----	-----	ns	ns	ns	ns	
Epietiocholanolone, C		ns	ns	0.811 **/**	0.762 **/**	ns	ns	-----	-----	0.853 **/*	0.802 **/**	
5β-Androstane-3α,17β-diol, C		ns	ns	0.857 **/**	0.838 **/**	ns	ns	0.91 **/**	0.771 **/**	-----	-----	

Note: Comparison between PG+ rats (n = 16) and PG− rats (n = 27). Values are correlation coefficients with a shared predictive component. Statistical significance is reported in the format OPLS/MR, where symbols before the slash refer to OPLS and symbols after the slash refer to multiple regression (MR). * *p* < 0.05, ** *p* < 0.01; ns, not significant.

## Data Availability

The datasets generated and analyzed during the current study are not publicly available due to their size and raw GC-MS/MS format but are available from the corresponding author upon reasonable request.

## Supplementary Materials

The following supporting information can be downloaded at: https://www.mdpi.com/article/10.3390/ijms27052506/s1.
