# Supplementary material for: Pregnanolone Glutamate: A Dual-Fate Delivery System for Neuroactive Steroids in Perinatal Focal Cerebral Ischemia
Source: Int J Mol Sci. 2026 Mar 9;27(5):2506. doi: 10.3390/ijms27052506 (PMC12985710; doi:10.3390/ijms27052506)
Supplement: Supplementary file 1 [file ijms-27-02506-s001.zip › Table S1.pdf]

Table S1. Pearson's correlation matrix of 5β-steroids in the serum of PG+ rats.

		Pregnanolone	Pregnanolone, C	Epipregnanolone	Epipregnanolone, C	17-Hydroxypregnanolone	17-Hydroxypregnanolone, C	5β,20α-Tetrahydroprogesterone	5β,20α-Tetrahydroprogesterone, C	5β-Pregnane-3α,20α-diol	5β-Pregnane-3α,20α-diol, C	5β-Pregnane-3β,20α-diol	5β-Pregnane-3α,17,20α-triol, C	Etiocholanolone	Etiocholanolone, C	Epietiocholanolone
		SERUM														
Pregnanolone	SERUM	1.0	0.8	0.8	0.5	0.3	0.5	0.9	-0.4	0.4	0.6	0.4	0.2	0.9	0.3	0.6
Pregnanolone, C		0.8	1.0	0.9	0.6	0.1	0.4	0.7	0.2	0.3	0.9	0.8	0.0	0.6	0.1	0.8
Epipregnanolone		0.8	0.9	1.0	0.7	-0.1	0.2	0.8	0.2	0.1	0.9	0.6	-0.2	0.7	-0.1	0.7
Epipregnanolone, C		0.5	0.6	0.7	1.0	-0.3	-0.2	0.7	0.4	-0.3	0.7	0.3	-0.3	0.6	-0.4	0.1
17-Hydroxypregnanolone		0.3	0.1	-0.1	-0.3	1.0	0.9	0.2	-0.7	1.0	-0.2	0.4	1.0	0.3	1.0	0.2
17-Hydroxypregnanolone, C		0.5	0.4	0.2	-0.2	0.9	1.0	0.3	-0.7	1.0	0.1	0.5	0.8	0.3	0.9	0.4
5β,20α-Tetrahydroprogesterone		0.9	0.7	0.8	0.7	0.2	0.3	1.0	-0.3	0.3	0.5	0.5	0.2	1.0	0.1	0.4
5β,20α-Tetrahydroprogesterone, C		-0.4	0.2	0.2	0.4	-0.7	-0.7	-0.3	1.0	-0.7	0.5	0.1	-0.7	-0.4	-0.7	-0.1
5β-Pregnane-3α,20α-diol		0.4	0.3	0.1	-0.3	1.0	1.0	0.3	-0.7	1.0	0.0	0.5	0.9	0.3	1.0	0.4
5β-Pregnane-3α,20α-diol, C		0.6	0.9	0.9	0.7	-0.2	0.1	0.5	0.5	0.0	1.0	0.7	-0.2	0.4	-0.1	0.7
5β-Pregnane-3β,20α-diol		0.4	0.8	0.6	0.3	0.4	0.5	0.5	0.1	0.5	0.7	1.0	0.4	0.4	0.5	0.7
5β-Pregnane-3α,17,20α-triol, C		0.2	0.0	-0.2	-0.3	1.0	0.8	0.2	-0.7	0.9	-0.2	0.4	1.0	0.2	1.0	0.1
Etiocholanolone		0.9	0.6	0.7	0.6	0.3	0.3	1.0	-0.4	0.3	0.4	0.4	0.2	1.0	0.2	0.4
Etiocholanolone, C		0.3	0.1	-0.1	-0.4	1.0	0.9	0.1	-0.7	1.0	-0.1	0.5	1.0	0.2	1.0	0.3
Epietiocholanolone		0.6	0.8	0.7	0.1	0.2	0.4	0.4	-0.1	0.4	0.7	0.7	0.1	0.4	0.3	1.0

Note: n = 6 (subset with complete body material collection). Significant correlations ($p < 0.05$) are highlighted with a yellow background. Strong positive correlations ($r > 0.7$) are in red; strong negative correlations ($r < -0.7$) are in green. C = conjugated steroid.
