# Supplementary material for: Pregnanolone Glutamate: A Dual-Fate Delivery System for Neuroactive Steroids in Perinatal Focal Cerebral Ischemia
Source: Int J Mol Sci. 2026 Mar 9;27(5):2506. doi: 10.3390/ijms27052506 (PMC12985710; doi:10.3390/ijms27052506)
Supplement: Supplementary file 1 [file ijms-27-02506-s001.zip › Table S2.pdf]

Table S2. Pearson's correlation matrix of 5β-steroids in the right hippocampus of PG+ rats.

		Pregnanolone	Pregnanolone, C	Epipregnanolone, C	17-Hydroxypregnanolone	17-Hydroxypregnanolone, C	5β,20α-Tetrahydroprogesterone	5β,20α-Tetrahydroprogesterone, C	5β-Pregnane-3α,20α-diol	5β-Pregnane-3α,20α-diol, C	5β-Pregnane-3β,20α-diol	5β-Pregnane-3β,20α-diol, C	5β-Pregnane-3α,17,20α-triol	Etiocholanolone	Etiocholanolone, C	Epietiocholanolone, C	5β-Androstane-3α,17β-diol, C
		RIGHT HIPPOCAMPUS															
Pregnanolone	RIGHT HIPPOCAMPUS	1.0	-0.4	-0.2	0.6	-0.6	0.8	0.6	0.9	-0.5	0.6	-0.1	0.0	0.0	-0.6	-0.1	-0.3
Pregnanolone, C		-0.4	1.0	0.5	-0.6	0.8	-0.6	-0.6	-0.3	0.9	-0.5	0.4	-0.3	-0.5	0.6	0.3	0.4
Epipregnanolone, C		-0.2	0.5	1.0	-0.5	0.5	-0.3	0.2	-0.4	0.3	-0.2	0.6	-0.5	-0.5	0.7	0.8	0.7
17-Hydroxypregnanolone		0.6	-0.6	-0.5	1.0	-0.5	0.6	0.3	0.6	-0.5	0.7	-0.3	0.6	0.6	-0.6	-0.5	-0.5
17-Hydroxypregnanolone, C		-0.6	0.8	0.5	-0.5	1.0	-0.7	-0.6	-0.6	0.8	-0.3	0.5	0.1	-0.3	0.8	0.4	0.6
5β,20α-Tetrahydroprogesterone		0.8	-0.6	-0.3	0.6	-0.7	1.0	0.6	0.7	-0.7	0.6	-0.1	0.1	0.2	-0.6	-0.2	-0.4
5β,20α-Tetrahydroprogesterone, C		0.6	-0.6	0.2	0.3	-0.6	0.6	1.0	0.5	-0.7	0.3	0.0	-0.3	-0.2	-0.3	0.2	0.0
5β-Pregnane-3α,20α-diol		0.9	-0.3	-0.4	0.6	-0.6	0.7	0.5	1.0	-0.4	0.5	-0.1	0.1	0.0	-0.7	-0.3	-0.4
5β-Pregnane-3α,20α-diol, C		-0.5	0.9	0.3	-0.5	0.8	-0.7	-0.7	-0.4	1.0	-0.5	0.3	-0.1	-0.3	0.6	0.1	0.3
5β-Pregnane-3β,20α-diol		0.6	-0.5	-0.2	0.7	-0.3	0.6	0.3	0.5	-0.5	1.0	0.1	0.5	0.5	-0.2	0.0	-0.1
5β-Pregnane-3β,20α-diol, C		-0.1	0.4	0.6	-0.3	0.5	-0.1	0.0	-0.1	0.3	0.1	1.0	-0.2	-0.3	0.4	0.3	0.5
5β-Pregnane-3α,17,20α-triol		0.0	-0.3	-0.5	0.6	0.1	0.1	-0.3	0.1	-0.1	0.5	-0.2	1.0	0.7	-0.1	-0.3	-0.3
Etiocholanolone		0.0	-0.5	-0.5	0.6	-0.3	0.2	-0.2	0.0	-0.3	0.5	-0.3	0.7	1.0	-0.3	-0.3	-0.4
Etiocholanolone, C		-0.6	0.6	0.7	-0.6	0.8	-0.6	-0.3	-0.7	0.6	-0.2	0.4	-0.1	-0.3	1.0	0.8	0.8
Epietiocholanolone, C		-0.1	0.3	0.8	-0.5	0.4	-0.2	0.2	-0.3	0.1	0.0	0.3	-0.3	-0.3	0.8	1.0	0.8
5β-Androstane-3α,17β-diol, C		-0.3	0.4	0.7	-0.5	0.6	-0.4	0.0	-0.4	0.3	-0.1	0.5	-0.3	-0.4	0.8	0.8	1.0

Note: n=16. Free epipregnanolone was excluded due to high inter-individual variability (CV≈82%) and lack of statistical significance ($p>0.05$ vs. PG− group). See Table 5 for tissue distribution values in a subset (n=6). Significant correlations ($p<0.05$) are highlighted in yellow. Strong positive correlations ($r>0.7$) are in red; strong negative correlations ($r<-0.7$) are in green. C=conjugated steroid.
