# Supplementary material for: Pregnanolone Glutamate: A Dual-Fate Delivery System for Neuroactive Steroids in Perinatal Focal Cerebral Ischemia
Source: Int J Mol Sci. 2026 Mar 9;27(5):2506. doi: 10.3390/ijms27052506 (PMC12985710; doi:10.3390/ijms27052506)
Supplement: Supplementary file 1 [file ijms-27-02506-s001.zip › Table S6.pdf]

Table S6. Pearson's correlation matrix of 5β-steroids between the serum and right hippocampus of PG-rats.

RIGHT HIPPOCAMPUS \ SERUM	Pregnanolone	Pregnanolone, C	Epipregnanolone	Epipregnanolone, C	17-Hydroxypregnanolone	17-Hydroxypregnanolone, C	5β,20α-Tetrahydroprogesterone	5β,20α-Tetrahydroprogesterone, C	5β-Pregnane-3α,20α-diol	5β-Pregnane-3α,20α-diol, C	5β-Pregnane-3β,20α-diol	5β-Pregnane-3α,17,20α-triol, C	Etiocholanolone	Etiocholanolone, C	Epietiocholanolone
Pregnanolone	0.6	0.3	-0.1	0.3	0.4	0.0	-0.3	-0.1	0.4	0.3	0.0	-0.2	0.1	0.0	-0.3
Pregnanolone, C	0.0	0.1	-0.2	0.0	0.0	-0.2	0.0	0.3	-0.4	0.1	0.4	0.0	0.0	0.1	0.0
Epipregnanolone	0.2	0.3	0.3	-0.1	0.1	0.3	0.3	0.0	0.3	-0.3	-0.3	0.0	-0.3	0.0	-0.1
Epipregnanolone, C	-0.1	0.1	0.1	-0.4	-0.2	-0.4	0.1	0.1	-0.3	0.3	0.5	0.4	0.0	-0.2	-0.1
17-Hydroxypregnanolone	0.5	0.5	-0.1	0.2	0.4	0.1	-0.1	-0.3	0.2	0.1	-0.5	-0.3	-0.2	0.1	-0.2
17-Hydroxypregnanolone, C	0.0	0.2	-0.2	0.1	-0.1	0.2	-0.1	0.3	0.0	0.1	0.0	0.2	0.2	-0.1	0.0
5β,20α-Tetrahydroprogesterone	0.3	0.3	0.2	0.0	0.1	0.0	0.1	-0.1	0.5	-0.4	0.0	-0.1	-0.6	0.0	-0.4
5β,20α-Tetrahydroprogesterone, C	0.1	0.1	-0.2	0.0	-0.1	-0.2	-0.2	0.2	-0.2	0.0	0.2	0.1	-0.1	-0.1	0.0
5β-Pregnane-3α,20α-diol	0.2	0.2	0.4	0.2	0.1	0.3	0.1	0.3	0.5	-0.4	-0.1	0.0	-0.3	-0.2	0.0
5β-Pregnane-3α,20α-diol, C	0.0	0.2	-0.1	0.3	0.0	0.3	0.0	0.2	0.3	0.1	-0.1	0.3	0.3	0.0	0.1
5β-Pregnane-3β,20α-diol	0.0	-0.1	-0.1	0.4	-0.3	0.1	0.2	0.6	0.1	0.1	0.4	0.3	0.3	0.0	0.3
5β-Pregnane-3β,20α-diol, C	0.1	0.2	-0.3	0.0	-0.2	-0.3	0.0	0.0	-0.3	0.2	0.2	0.2	0.0	-0.1	-0.2
5β-Pregnane-3α,17,20α-triol	0.2	0.2	0.0	0.1	-0.2	0.2	0.0	0.2	0.3	-0.1	-0.1	-0.1	-0.2	0.0	-0.1
Etiocholanolone	0.3	0.3	0.1	0.2	-0.1	0.0	0.1	0.0	0.5	-0.1	0.1	0.2	-0.1	-0.2	0.0
Etiocholanolone, C	0.1	0.1	0.0	0.0	-0.1	-0.3	-0.2	-0.1	0.0	0.0	0.3	-0.2	0.0	0.1	0.2
Epietiocholanolone	0.1	0.2	0.1	0.0	0.0	-0.3	0.6	-0.2	0.1	-0.2	0.1	0.1	-0.2	-0.1	0.0
Epietiocholanolone, C	0.0	0.1	0.0	-0.3	-0.2	-0.2	-0.3	0.0	0.0	0.3	0.2	0.1	0.2	0.0	0.1
5β-Androstane-3α,17β-diol, C	0.4	0.5	-0.2	0.2	0.0	0.1	-0.3	0.1	0.4	0.1	0.3	0.0	-0.1	0.1	-0.2

Note: n = 21 (subset with complete body material collection). Significant correlations ($p < 0.05$) are highlighted with a yellow background. Strong positive correlations ($r > 0.7$) are in red; strong negative correlations ($r < -0.7$) are in green. C = conjugated steroid.
