# Supplementary material for: Pregnanolone Glutamate: A Dual-Fate Delivery System for Neuroactive Steroids in Perinatal Focal Cerebral Ischemia
Source: Int J Mol Sci. 2026 Mar 9;27(5):2506. doi: 10.3390/ijms27052506 (PMC12985710; doi:10.3390/ijms27052506)
Supplement: Supplementary file 1 [file ijms-27-02506-s001.zip › Table S8.pdf]

Table S8. Pearson's correlation matrix of 5α-steroids in the right hippocampus of PG+ rats.

RIGHT HIPPOCAMPUS	5α-Dihydroprogesterone	Allopregnanolone	Allopregnanolone, C	Isopregnanolone	Isopregnanolone, C	17-Hydroxyallopregnanolone	17-Hydroxyallopregnanolone, C	5α,20α-Tetrahydroprog.	5α,20α-Tetrahydroprog., C	5α-Pregnane-3α,20α-diol	5α-Pregnane-3α,20α-diol, C	5α-Pregnane-3β,20α-diol	5α-Pregnane-3β,20α-diol, C	5α-Pregnane-3α,17,20α-triol	Androsterone	Androsterone, C	Epiandrosterone	Epiandrosterone, C	5α-Androstane-3α,17β-diol	5α-Androstane-3α,17β-diol, C	5α-Androstane-3β,17β-diol	5α-Androstane-3β,17β-diol, C	11β-Hydroxyandrosterone, C	11β-Hydroxyepiandrosterone, C
5α-Dihydroprogesterone	1.0	0.6	-0.4	-0.2	-0.7	0.5	-0.6	0.6	0.3	0.5	-0.5	0.2	-0.3	0.3	0.6	-0.4	0.6	-0.5	0.5	-0.2	0.1	-0.5	0.3	-0.2
Allopregnanolone	0.6	1.0	0.3	0.0	-0.5	0.7	-0.4	0.8	0.8	0.9	0.0	0.6	-0.2	0.4	0.6	-0.4	0.5	-0.5	0.4	-0.5	0.3	-0.4	-0.1	-0.3
Allopregnanolone, C	-0.4	0.3	1.0	0.2	0.5	0.1	0.5	-0.1	0.2	0.4	0.7	0.4	0.5	-0.1	-0.4	0.3	-0.1	0.4	-0.1	0.3	0.2	0.4	-0.2	0.1
Isopregnanolone	-0.2	0.0	0.2	1.0	0.6	-0.1	0.3	-0.2	-0.2	0.3	0.5	0.0	0.5	0.0	0.2	0.4	0.0	0.6	0.4	0.1	0.6	0.5	-0.1	0.2
Isopregnanolone, C	-0.7	-0.5	0.5	0.6	1.0	-0.5	0.7	-0.6	-0.5	-0.3	0.4	-0.4	0.7	-0.3	-0.5	0.7	-0.4	0.9	-0.3	0.6	0.3	0.8	0.1	0.5
17-Hydroxyallopregnanolone	0.5	0.7	0.1	-0.1	-0.5	1.0	-0.1	0.6	0.7	0.8	0.3	0.6	-0.2	0.5	0.3	-0.6	0.6	-0.6	0.2	-0.4	0.0	-0.5	-0.1	-0.6
17-Hydroxyallopregnanolone, C	-0.6	-0.4	0.5	0.3	0.7	-0.1	1.0	-0.6	-0.4	-0.2	0.7	-0.2	0.7	-0.1	-0.4	0.5	-0.3	0.7	-0.3	0.5	0.1	0.7	0.0	0.3
5α,20α-Tetrahydroprog.	0.6	0.8	-0.1	-0.2	-0.6	0.6	-0.6	1.0	0.8	0.7	-0.2	0.6	-0.5	0.6	0.5	-0.6	0.5	-0.7	0.3	-0.6	0.3	-0.6	0.1	-0.3
5α,20α-Tetrahydroprog., C	0.3	0.8	0.2	-0.2	-0.5	0.7	-0.4	0.8	1.0	0.7	0.1	0.8	-0.4	0.3	0.3	-0.6	0.3	-0.6	0.1	-0.6	0.1	-0.6	-0.2	-0.5
5α-Pregnane-3α,20α-diol	0.5	0.9	0.4	0.3	-0.3	0.8	-0.2	0.7	0.7	1.0	0.4	0.7	0.1	0.3	0.5	-0.3	0.5	-0.3	0.4	-0.4	0.5	-0.2	-0.1	-0.3
5α-Pregnane-3α,20α-diol, C	-0.5	0.0	0.7	0.5	0.4	0.3	0.7	-0.2	0.1	0.4	1.0	0.3	0.6	0.0	-0.2	0.2	-0.1	0.3	-0.1	0.1	0.3	0.4	-0.3	-0.1
5α-Pregnane-3β,20α-diol	0.2	0.6	0.4	0.0	-0.4	0.6	-0.2	0.6	0.8	0.7	0.3	1.0	-0.2	0.1	0.0	-0.4	0.5	-0.4	0.3	-0.4	0.1	-0.4	-0.1	-0.4
5α-Pregnane-3β,20α-diol, C	-0.3	-0.2	0.5	0.5	0.7	-0.2	0.7	-0.5	-0.4	0.1	0.6	-0.2	1.0	-0.3	-0.3	0.7	-0.3	0.8	-0.1	0.7	0.5	0.9	0.4	0.6
5α-Pregnane-3α,17,20α-triol	0.3	0.4	-0.1	0.0	-0.3	0.5	-0.1	0.6	0.3	0.3	0.0	0.1	-0.3	1.0	0.6	-0.2	0.5	-0.4	0.4	-0.5	0.3	-0.4	0.1	-0.1
Androsterone	0.6	0.6	-0.4	0.2	-0.5	0.3	-0.4	0.5	0.3	0.5	-0.2	0.0	-0.3	0.6	1.0	-0.2	0.4	-0.4	0.4	-0.7	0.4	-0.5	0.0	-0.2
Androsterone, C	-0.4	-0.4	0.3	0.4	0.7	-0.6	0.5	-0.6	-0.6	-0.3	0.2	-0.4	0.7	-0.2	-0.2	1.0	-0.3	0.8	0.0	0.6	0.5	0.8	0.4	0.8
Epiandrosterone	0.6	0.5	-0.1	0.0	-0.4	0.6	-0.3	0.5	0.3	0.5	-0.1	0.5	-0.3	0.5	0.4	-0.3	1.0	-0.4	0.7	-0.4	0.3	-0.4	0.0	-0.4
Epiandrosterone, C	-0.5	-0.5	0.4	0.6	0.9	-0.6	0.7	-0.7	-0.6	-0.3	0.3	-0.4	0.8	-0.4	-0.4	0.8	-0.4	1.0	-0.1	0.7	0.4	1.0	0.3	0.7
5α-Androstane-3α,17β-diol	0.5	0.4	-0.1	0.4	-0.3	0.2	-0.3	0.3	0.1	0.4	-0.1	0.3	-0.1	0.4	0.4	0.0	0.7	-0.1	1.0	-0.3	0.4	-0.2	0.0	0.0
5α-Androstane-3α,17β-diol, C	-0.2	-0.5	0.3	0.1	0.6	-0.4	0.5	-0.6	-0.6	-0.4	0.1	-0.4	0.7	-0.5	-0.7	0.6	-0.4	0.7	-0.3	1.0	0.0	0.7	0.4	0.6
5α-Androstane-3β,17β-diol	0.1	0.3	0.2	0.6	0.3	0.0	0.1	0.3	0.1	0.5	0.3	0.1	0.5	0.3	0.4	0.5	0.3	0.4	0.4	0.0	1.0	0.3	0.4	0.4
5α-Androstane-3β,17β-diol, C	-0.5	-0.4	0.4	0.5	0.8	-0.5	0.7	-0.6	-0.6	-0.2	0.4	-0.4	0.9	-0.4	-0.5	0.8	-0.4	1.0	-0.2	0.7	0.3	1.0	0.3	0.7
11β-Hydroxyandrosterone, C	0.3	-0.1	-0.2	-0.1	0.1	-0.1	0.0	0.1	-0.2	-0.1	-0.3	-0.1	0.4	0.1	0.0	0.4	0.0	0.3	0.0	0.4	0.4	0.3	1.0	0.6
11β-Hydroxyepiandrosterone, C	-0.2	-0.3	0.1	0.2	0.5	-0.6	0.3	-0.3	-0.5	-0.3	-0.1	-0.4	0.6	-0.1	-0.2	0.8	-0.4	0.7	0.0	0.6	0.4	0.7	0.6	1.0

Note: n = 16. Significant correlations ($p < 0.05$) are highlighted with a yellow background. Strong positive correlations ($r > 0.7$) are in red; strong negative correlations ($r < –0.7$) are in green. C = conjugated steroid.
